# Mechanistic Insights Into Color and Texture Stability of Frozen Tropical Vegetables: Processing, Modeling, and Preservation Strategies

**DOI:** 10.1002/fsn3.71895

**Published:** 2026-06-01

**Authors:** Muhammad Muntasir Mahmud, Md. Suhel Mia, Md. Hassan Bin Nabi, Abid Hassan Arnab, Wahidu Zzaman

**Affiliations:** ^1^ Department of Food Engineering and Tea Technology Shahjalal University of Science and Technology Sylhet Bangladesh

**Keywords:** color stability, frozen storage, ice crystal formation, pigment oxidation, texture degradation, tropical vegetables

## Abstract

Freezing is the most effective preservation technique for maintaining the quality and nutritional integrity of vegetables, particularly in tropical regions where postharvest losses are high. However, during long‐term frozen storage, undesirable changes in color and texture often occur, reducing consumer acceptability and export quality. These alterations are linked to biochemical and physical mechanisms, including pigment oxidation, enzymatic activity, ice crystal formation, and cellular structure collapse. This review critically summarizes recent research on color and texture stability in frozen tropical vegetables such as taro stolon, lablab bean seeds, okra, and green beans, which were selected due to their economic importance in tropical regions, high moisture content, and pronounced susceptibility to postharvest quality deterioration during freezing and storage. The mechanistic insights are discussed in relation to processing factors such as blanching, freezing rate, and storage temperature (−20°C), along with advanced modeling approaches for predicting quality degradation. The review also highlights the role of natural antioxidants, pretreatments, and novel freezing technologies in improving color and textural stability, providing a foundation for future industrial and mechanistic research. Despite extensive research, an integrated mechanistic link between physicochemical changes, microstructural damage, and predictive modeling in tropical frozen vegetables under dynamic cold chains remains limited. This review synthesizes degradation pathways and combines kinetic, spectroscopic, and data‐driven models for comprehensive quality prediction.

## Graphical abstract

This review elucidates the mechanistic pathways governing color and texture degradation in frozen tropical vegetables, emphasizing pigment oxidation, enzymatic softening, and ice crystal‐induced structural damage. It integrates kinetic, Arrhenius, RSM, and machine‐learning models with advanced preservation strategies to optimize freezing processes, enhance quality retention, and improve cold‐chain stability in tropical vegetable systems.

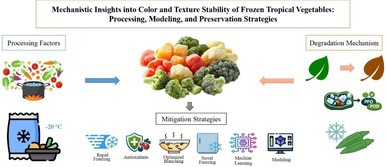

## 1 Introduction

Tropical vegetables constitute an essential component of food security, nutrition, and agricultural economies in South and Southeast Asia, where they contribute significantly to dietary diversity, micronutrient intake, and export revenue. Vegetables such as taro stolon ( *Colocasia esculenta* ), lablab bean ( *Lablab purpureus* ), okra ( *Abelmoschus esculentus* ), and yardlong bean ( *Vigna unguiculata* subsp. *sesquipedalis*) are widely consumed and valued for their high contents of dietary fiber, vitamins, minerals, and bioactive compounds. However, the commercialization and long‐distance distribution of these commodities are constrained by their high moisture content, intense metabolic activity, and susceptibility to enzymatic and microbial spoilage, which result in rapid postharvest deterioration (Rahman et al. 2022).

Freezing is widely regarded as one of the most effective preservation strategies for extending the shelf life of tropical vegetables, as it markedly suppresses microbial growth and slows down biochemical reactions. Despite these advantages, maintaining quality attributes, particularly color and texture, during frozen storage remains a persistent challenge. Under conventional industrial conditions, frozen vegetables are typically stored at approximately −20°C, a temperature that is insufficient to completely arrest physicochemical and enzymatic degradation processes. As a result, frozen tropical vegetables often exhibit noticeable quality losses during storage and subsequent thawing, limiting consumer acceptance and market value (Billah, Rahman, et al. 2025; Langston et al. 2021). Color and texture are the primary quality attributes governing consumer perception of frozen vegetables. Color is closely associated with freshness and nutritional quality and is determined by the stability of plant pigments, including chlorophylls, carotenoids, and anthocyanins. Texture, on the other hand, reflects the structural integrity of plant tissues and is governed by cellular turgor pressure, the architecture of the cell wall matrix (cellulose, hemicellulose, and lignin), and the pectic network in the middle lamella, which is often reinforced by calcium cross‐linking. The degradation of color and texture during freezing and frozen storage is not driven by a single factor but results from the complex interplay of residual enzymatic activity, oxidative reactions, and physical damage induced by ice crystal formation and recrystallization (Sun et al. 2023).

The freezing rate and storage temperature history play a decisive role in determining the extent of structural damage in frozen vegetables. Slow freezing and temperature fluctuations during storage promote the formation and growth of large extracellular and intracellular ice crystals, which can rupture cell membranes and cell walls. This physical disruption leads to irreversible loss of cellular compartmentalization, resulting in reduced turgor, increased drip loss upon thawing, and pronounced softening of tissues. Moreover, membrane damage facilitates interactions between enzymes and their substrates that are normally segregated within intact cells, thereby accelerating pigment degradation, enzymatic browning, and other quality‐deteriorating reactions during frozen storage and thawing (Liao et al. 2024).

In recent years, advances in kinetic modeling, nondestructive analytical techniques, and data‐driven approaches have significantly enhanced the ability to monitor and predict quality changes in frozen vegetables. Color and texture degradation are frequently described using pseudo‐zero‐order or pseudo‐first‐order kinetic models, while temperature dependence is commonly characterized using Arrhenius‐type relationships to quantify activation energies and degradation rates. These models provide valuable insights into the sensitivity of quality attributes to processing and storage conditions and enable more realistic shelf life predictions under both static and dynamic cold‐chain scenarios (Giannakourou and Taoukis 2020; Rashvand et al. 2025). While Arrhenius‐based kinetic models provide interpretable parameters such as activation energy and degradation rate constants, they are limited in capturing nonlinear interactions and dynamic temperature fluctuations typical of real cold chains. In contrast, data‐driven approaches such as artificial neural networks and support vector regression can model complex, nonlinear relationships among multiple variables, including temperature history, moisture migration, and enzymatic activity. However, these models often require large datasets and lack mechanistic interpretability. Therefore, hybrid approaches integrating mechanistic kinetics with machine learning are increasingly recommended for robust and scalable quality prediction.

Furthermore, the integration of machine‐learning approaches such as artificial neural networks and support vector regression with noninvasive techniques like near‐infrared spectroscopy has opened new possibilities for real‐time quality assessment of frozen vegetables. These tools offer the potential to capture complex, nonlinear relationships between processing variables and quality outcomes, thereby supporting more precise control of freezing operations and cold‐chain management (Li et al. 2024; Jiang, Peng, et al. 2023). Despite these advances, a comprehensive synthesis of the mechanistic pathways governing color and texture degradation in frozen tropical vegetables, together with emerging analytical and predictive approaches, remains limited.

Therefore, this review aims to provide an integrated and mechanistic overview of color and texture stability in frozen tropical vegetables. The paper critically examines the physicochemical, enzymatic, and structural mechanisms underlying quality degradation, evaluates the role of freezing and blanching conditions, and highlights recent advances in kinetic modeling, spectroscopy, and machine‐learning‐based quality prediction. By consolidating current knowledge and identifying existing research gaps, this review seeks to support the development of improved preservation strategies and quality management systems for frozen tropical vegetables.

## 2 Overview of Quality Degradation

**FIGURE 1:**
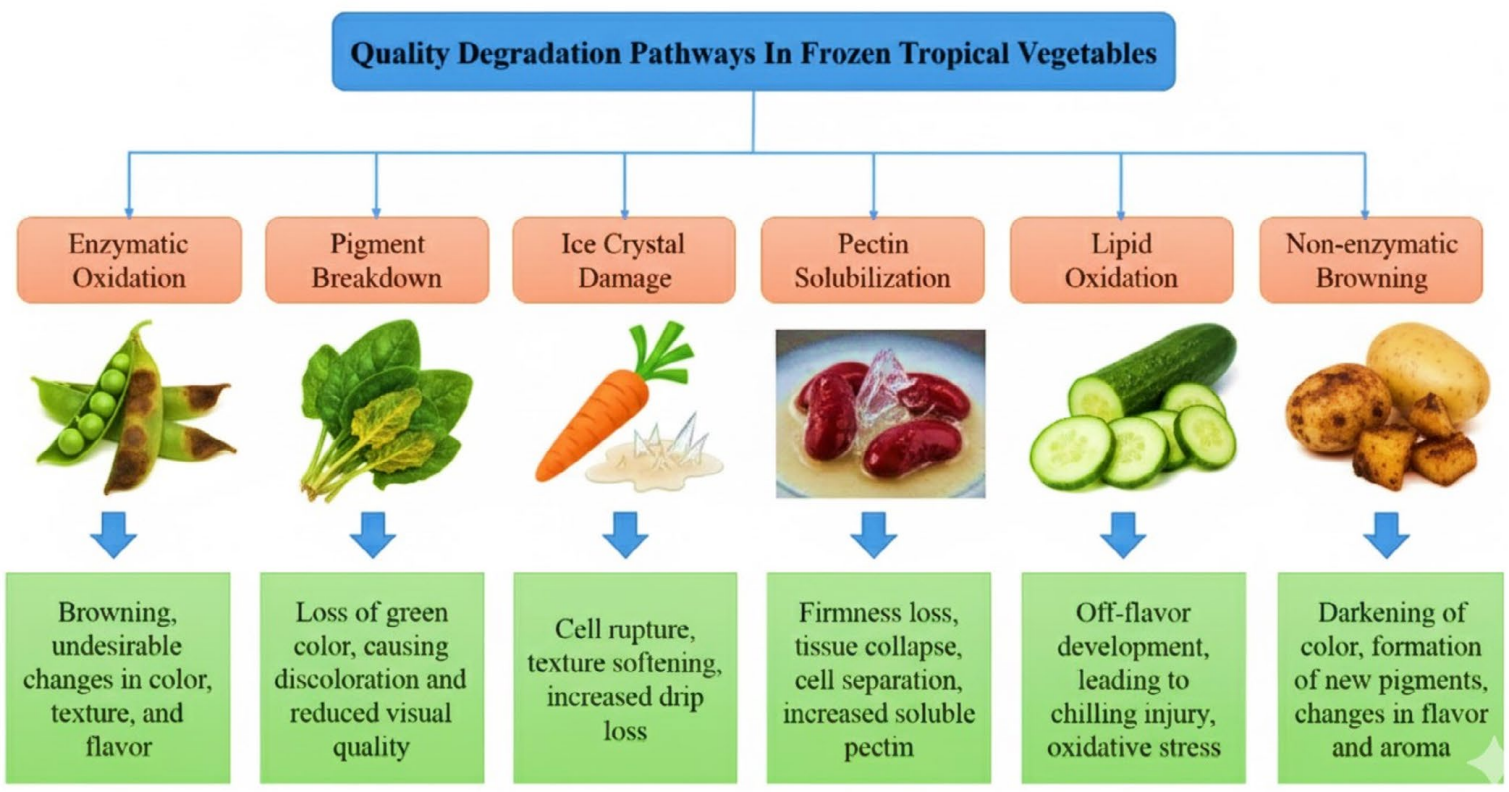
Quality degradation pathways in frozen vegetables.

Frozen storage effectively slows chemical reactions, reduces microbial activity, and extends the shelf life of tropical vegetables (Rahman et al. 2022; Zhao et al. 2025). However, despite the protective effects of low temperatures, quality degradation continues to occur during prolonged storage due to intertwined physical, biochemical, and chemical mechanisms. These changes manifest as color alteration, textural softening, nutrient loss, and sensory decline, challenging frozen quality maintenance and export acceptability (Figure 1). The formation and growth of ice crystals are fundamental drivers of physical degradation in frozen vegetables. As the water in plant tissues freezes, it first forms extracellular ice crystals, increasing the solute concentration in the remaining unfrozen fraction and causing osmotic cellular dehydration. This dehydration reduces turgor pressure, a critical determinant of vegetable firmness and crispness. Slow cooling rates promote the aggregation of fewer but larger ice crystals, which can mechanically disrupt cell walls and membranes, leading to irreversible cellular disintegration and textural softening. In contrast, rapid freezing produces a multitude of small, more uniformly distributed crystals, which generally cause less physical damage but remain a risk factor during temperature fluctuation in storage (Zhan et al. 2018). Quantitatively, slow freezing (0.1°C–1°C/min) typically produces ice crystals exceeding 50–100 μm in diameter, whereas rapid freezing (> 10°C/min) results in smaller crystals (< 10–20 μm), which are less destructive to cellular structures. Ice recrystallization kinetics during storage can further increase crystal size by 2–5 times, significantly correlating with increased drip loss (10%–25%) and firmness reduction. These quantitative relationships highlight the importance of controlling both freezing rate and storage temperature stability (Zhan et al. 2018). During extended frozen storage, ice recrystallization further exacerbates cellular damage. Recrystallization magnifies mechanical stress on microstructures, progressively weakening cellular integrity and accelerating softening. This process is particularly detrimental for high‐water‐content tropical vegetables, as expanded ice crystals compress and rupture the primary and middle lamellae of cell walls, producing tissues that are mushy upon thawing (Vicent et al. 2020).

Plant tissues inherently contain enzymes such as polyphenol oxidase (PPO), peroxidase (POD), pectin methylesterase (PME), and polygalacturonase (PG), which remain partially active even at subzero temperatures. The cold concentration effect where freeze‐concentrated solutes and reduced water activity bring enzymes and substrates into closer proximity can permit slow but meaningful biochemical reactions. PPO and POD, in particular, catalyze oxidation of phenolic compounds leading to enzymatic browning, while pectinolytic enzymes degrade pectin networks, contributing to cell wall loosening and textural deterioration. As molecular mobility is reduced but not eliminated at frozen temperatures, these reactions continue at a diminished pace yet accumulate over long storage durations, contributing to color and texture changes (Van der Sman 2020; Terefe et al. 2013). As illustrated in Figure 1, quality degradation in frozen tropical vegetables is governed by the simultaneous interaction of physical (ice crystal formation and recrystallization), biochemical (enzymatic activity), and chemical (oxidative reactions) processes. The figure highlights how these mechanisms are interdependent, with structural damage facilitating enzymatic and oxidative pathways that accelerate overall deterioration.

Chemical degradation processes, such as lipid oxidation and pigment breakdown, also contribute to quality loss. Oxidative reactions can occur due to residual oxygen in packaging or within tissues, even under frozen conditions, leading to degradation of pigments like chlorophylls and carotenoids. Oxidative cleavage of chlorophyll, for example, results in the formation of pheophytins, which alter the green color toward brownish hues, diminishing visual appeal. Although Maillard reactions are generally limited under frozen conditions due to low molecular mobility, they may occur during temperature abuse or thawing stages, contributing to undesirable color and flavor changes (Neri et al. 2020). Beyond color and texture, nutrient profiles and sensory qualities also change during frozen storage. Hydrophilic vitamins such as vitamin C and thiamin are susceptible to degradation during pre‐freezing treatments and extended storage, particularly when blanching protocols are not optimized or when enzymatic activity persists. Lipophilic nutrients, including β‐carotene, are more stable but can still decline under oxidative stress and prolonged storage. In addition, flavor volatile compounds can be lost or transformed, reducing overall palatability. While freezing slows most degradative pathways, multiple freeze–thaw cycles often encountered in variable cold chains exacerbate nutrient leaching, pigment oxidation, and textural collapse (Zhao et al. 2025).

Quality degradation in frozen tropical vegetables is not the result of single, isolated phenomena but rather an interconnected network of physical, biochemical, and chemical processes (Table 1). Ice crystal formation and recrystallization physically disrupt cell architecture, enabling biochemical reactions by bringing enzymes into contact with substrates and thereby amplifying pigment and structural polymer breakdown. Simultaneously, chemical oxidation contributes to both direct pigment loss and lipid peroxidation, affecting color, flavor, and membrane stability. These processes, while slowed by low temperatures, are cumulative over time and are highly sensitive to storage temperature stability, packaging atmosphere, pretreatments, and inherent tissue properties. Effective quality preservation, therefore, requires an integrated understanding of these mechanisms and targeted interventions across the processing and cold‐chain continuum (Matabura 2023). Table 1 summarizes the key degradation mechanisms affecting color and texture in frozen tropical vegetables, along with their underlying physicochemical drivers and resulting quality impacts.

**TABLE 1:** Major factors influencing color and texture degradation in frozen tropical vegetables during storage at −20°C.

Mechanism	Key cause	Effect on quality	Specific vegetable(s)	Recent mitigation strategies	References
Enzymatic oxidation (PPO)	Residual polyphenol oxidase (PPO) activity after incomplete blanching	Browning, color darkening, visible browning within 2–4 weeks	Brussels sprouts	Precision thermal blanching (90°C–100°C, 50–180 s) for > 90% PPO inactivation; blanching 80°C × 400 s optimal	Pérez‐Calderón et al. (2019)
Residual peroxidase (POD) activity	Incomplete enzyme inactivation during blanching; POD biphasic thermal behavior (heat‐labile and heat resistant isoforms)	Quality deterioration, color, chlorophyll pheophytisation (vivid green → olive brown); sensory quality loss	Green beans	Extended blanching (80°C–95°C, 3–5 min); blanching at 82°C × 3.5 min yields highest sensory scores (> 7/9 panel score)	Lee et al. (1988), Martins and Silva (2004)
Lipoxygenase activity (LOX)	Lipid oxidation by residual LOX enzymes; formation of volatile organic compounds (VOCs) from polyunsaturated fatty acids (PUFA)	Off‐flavor formation (grassy, green‐like C6 and C9 aldehydes from PUFA oxidation); aroma loss within 3–6 months	Brussels sprouts	Thermal blanching (85°C–90°C, 2–3 min for 90% LOX inactivation); pulsed electric field (PEF) pretreatment to control enzyme‐substrate interactions; nitrogen gas packaging	Delbaere et al. (2022)
Chlorophyllase enzyme activity	Chlorophyllase‐mediated degradation of chlorophyll a and b; pH sensitivity; light exposure and temperature fluctuations	Chlorophyll degradation and pheophytin formation (olive green → grayish‐brown)	Mulberry leaves ( *Morus alba* L.)	Alkaline blanching water, sodium bicarbonate; calcium chloride; low‐light storage (dark/opaque packaging)	Zhao et al. (2024)
Carotenoid degradation	Oxidative cleavage of carotenoids by singlet oxygen; thermal degradation during blanching (5%–15% loss); lycopene more labile than β‐carotene	Loss of yellow‐orange pigments, color fading; β‐carotene retention > 95% during processing; lycopene degradation	Carrots ( *Daucus carota* L.)	Vacuum packaging + inert gas flush (N_2_); isochoric freezing (reduces ice crystal size, carotenoid oxidation); antioxidant pretreatment	Behsnilian and Mayer‐Miebach (2017)
Nonenzymatic browning (Maillard reaction)	Reducing sugars + amino acids + extended storage at elevated subzero temps; exacerbated by temperature fluctuations	Darkening (ΔE > 5 units in 12 months at fluctuating temps); altered flavor profile (caramel‐like off‐flavors); formation of advanced glycation end products (AGEs)	Sweet corn ( *Zea mays* L. var. saccharata)	Moisture control; hermetic sealing + desiccant packs; constant −20°C ± 2°C storage; controlled atmosphere	Sun et al. (2023)
Oxidative browning (ascorbic acid degradation)	Autooxidation of ascorbic acid to dehydroascorbic acid (DHAA); catalyzed by trace metals (Cu^2+^, Fe^3+^); first‐order kinetics, Ea = 12.5–45 kJ/mol (low activation energy)	Loss of ascorbic acid (50%–80% loss in 12 months at −20°C); color shift toward brown–gray; reduced antioxidant capacity (ORAC) by 40%–60%	Green beans ( *Phaseolus vulgaris* L.)	Metal chelating agents; oxygen scavenging films; vacuum freezing; blanching in ascorbic acid solution (1%–2% AA dip pre‐freeze)	Martins and Silva (2004)
Texture degradation: ice crystal formation	Formation of large ice crystals during slow freezing; crystal penetration through cell walls; recrystallization during temperature fluctuations	Loss of firmness; increased drip loss upon thawing; cell structure collapse, mushy texture	Carrots ( *Daucus carota* L.)	Rapid freezing (< −40°C within 30 min); liquid nitrogen freezing (LNF); high‐pressure freezing; ultrasound‐assisted freezing	Paciulli et al. (2016)
Cell wall degradation: pectin‐cellulose network	Residual pectinase and cellulase enzymes degrade cell wall polysaccharides; calcium deficiency promotes softening; freeze–thaw cycles rupture cell walls	Firmness loss; increased cellular leakage; reduced crispness; sogginess upon rehydration; pectin degradation	Green beans ( *Phaseolus vulgaris* L.)	Calcium chloride pretreatment; enzyme blanching optimization; avoidance of temperature fluctuations	Lee et al. (1988), Martins and Silva (2004)
Enzymatic browning during thawing	Compartmentalization loss during freeze–thaw cycle; PPO and phenolic substrates contact oxygen upon thaw; enzyme reactivation in unfrozen liquid phase	Rapid browning; darkening of surface tissue; progressive browning; browning index increases	Green peppers ( *Capsicum annuum* L.)	Slow thawing protocols; controlled atmosphere thawing; osmotic dehydration pre‐thaw; anti‐browning dip post‐thaw (ascorbic acid 1%, calcium lactate 0.5%)	Liang et al. (2025)
Chlorophyll → pheophytin conversion	Loss of central Mg^2+^ from chlorophyll molecule via acid catalysis; chlorophyllase enzyme activity increases; unfrozen liquid phase promotes Mg^2+^ leaching	Gray‐brown discoloration, olive green → yellowish‐brown hue; pheophytin formation	Mulberry leaves ( *Morus alba* L.)	Alkaline blanching water; magnesium salt additives (MgCl_2_ 0.1%–0.2%); vacuum + inert gas packaging; pH‐buffered storage environment; light exclusion (opaque/chromatic filters)	Zhao et al. (2024)
Lipid peroxidation	Oxidation of polyunsaturated fatty acids (PUFA) catalyzed by LOX, POD, PPO, or nonenzymatic free radicals; malondialdehyde (MDA) formation; secondary oxidation products accumulate	Off‐odor development; altered sensory attributes; loss of volatile compounds; rancidity in long‐term storage; TBA values increase 0.5–1.2 mg MDA/kg in 12 months	Peas ( *Pisum sativum* L.), spinach ( *Spinacia oleracea* L.), soybeans ( *Glycine max* L.)	Natural antioxidants, rosemary extract, ascorbic acid; enzyme blanching (85°C–95°C, 2–3 min for ≥ 80% LOX inactivation); vacuum packaging; reduced O_2_	Neri et al. (2020), Bhowmik et al. (2023)
Anthocyanin degradation	Thermal and oxidative degradation; pH sensitivity; condensation with PPO‐derived quinones; light‐induced oxidative cleavage	Color shift purple/red → brown; anthocyanin loss 40%–60%; reduced antioxidant activity; formation of brown polymeric compounds	Red cabbage ( *Brassica oleracea var. capitata* f. rubra), purple sweet corn	Acidification; antioxidant co‐pigmentation; light exclusion; controlled atmosphere storage; rapid freezing to < −40°C; SO_2_ treatment	Zhang et al. (2023)
Vitamin retention loss (A, C, E)	Enzymatic oxidation (PPO, POD); nonenzymatic oxidation; interaction with PPO/POD‐derived quinones; thermal damage during blanching (15%–25% loss); losses accelerate exponentially months 1–6	Reduction vitamin A, vitamin C, vitamin E; nutritional degradation; blanching alone causes 10%–30% loss before storage; vitamin C losses most significant	Broccoli ( *Brassica oleracea var. italica* ), carrots ( *Daucus carota* L.), green peas ( *Pisum sativum* L.), bell peppers ( *Capsicum annuum* L.), tomatoes ( *Solanum lycopersicum* L.)	Optimized blanching (70°C–85°C, 2–3 min); rapid cooling post‐blanch; vacuum/MAP packaging; antioxidant fortification; storage < −25°C if feasible; light/oxygen control	Rickman et al. (2007), Wang et al. (2022)
Freeze–thaw recrystallization	Temperature fluctuations during storage/transport; water sublimation; ice recrystallization (Ostwald ripening); repeated cycling accumulates damage	Formation of larger ice crystals; drip loss (15%–30% after thaw); surface dehydration (“freezer burn”); frost accumulation	Leafy greens (spinach, kale) especially sensitive; green beans, carrots show quantifiable frost accumulation	Vacuum‐sealed packaging with moisture barriers; desiccant packs (silica gel); hermetic sealing; temperature stability; active packaging with oxygen scavengers	Simoes (2025)
Cellulase & hemicellulase activity	Residual cellulase and hemicellulase enzymes degrade cell wall polysaccharides; enzyme activation during thawing in unfrozen liquid phase; incomplete inactivation by suboptimal blanching	Breakdown of structural integrity, loss of crispness; softening; drip loss; sogginess upon rehydration; polysaccharide degradation 30%–50% in unblanched samples	Green beans ( *Phaseolus vulgaris* L.) peas ( *Pisum sativum* L.) –turnip greens ( *Brassica rapa* L.)	Enzyme inactivation blanching; calcium fortification (0.5%–1.0% CaCl_2_); controlled thawing; edible coatings post‐thaw (chitosan 0.5%–1.0%, alginate); magnetic field during freezing	Martínez et al. (2012), Lee et al. (1988)

## 3 Color Degradation

**FIGURE 2:**
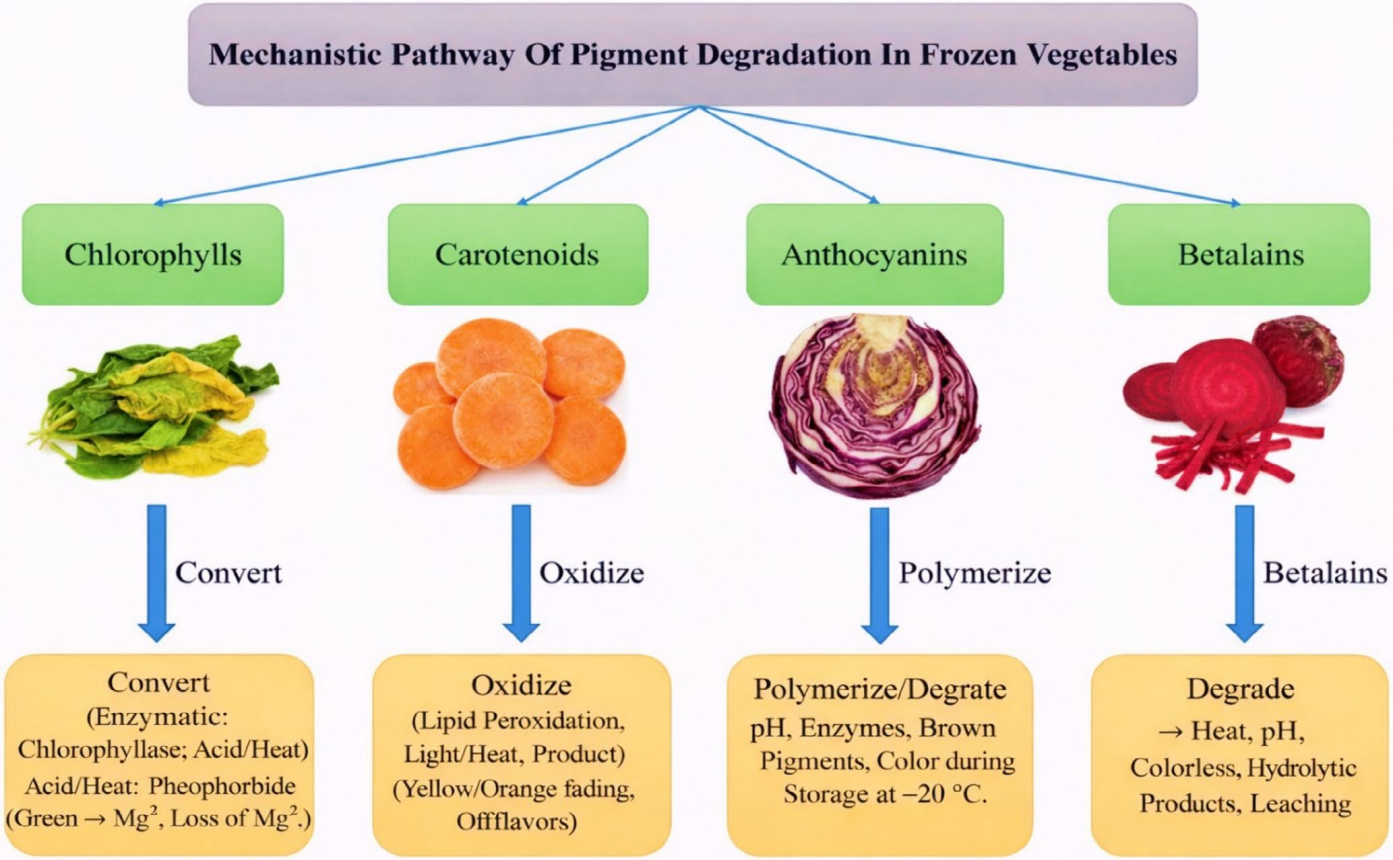
Pathway of pigment degradation in frozen vegetables.

Color stability is a critical indicator of the freshness, nutritional quality, and marketability of frozen tropical vegetables. Discoloration not only affects consumer acceptance but also reflects underlying biochemical and physical deterioration processes. In heterogeneous tissues such as taro stolon, lablab bean, okra, and green beans, differential sensitivity of pigment classes (chlorophylls, carotenoids, and anthocyanins) to freezing and storage stress leads to distinct degradation patterns under cold chain conditions (Table 2). The principal mechanisms responsible for pigment loss in frozen tropical vegetable tissues include chlorophyll degradation, carotenoid oxidation, and anthocyanin breakdown. Each is governed by interacting physical, biochemical, and chemical drivers that remain active, even if slowed, under subzero conditions (Figure 2). Freezing induces cellular disruption through ice crystal formation and solute concentration effects, which alter intracellular pH, release compartmentalized enzymes, and increase the contact between pigments and prooxidant species. In green tissues, chlorophyll destabilization is promoted by acidification and metal‐ion displacement, leading to pheophytin and pheophorbide formation and a progressive shift from bright green to olive‐brown hues. Carotenoids, although more structurally stable than chlorophylls, are highly susceptible to oxidative cleavage and isomerization reactions catalyzed by residual oxygen, free radicals, and lipoxygenase‐mediated lipid oxidation, resulting in fading of yellow–orange coloration and loss of provitamin activity. Anthocyanins in pigmented tropical vegetables are particularly sensitive to pH shifts, enzymatic oxidation, and polymerization reactions with phenolics and proteins, which produce browning and color dullness over storage time. Importantly, these pathways rarely occur in isolation; instead, they are coupled with temperature fluctuations, recrystallization‐driven membrane damage, and incomplete enzyme inactivation during blanching, creating microenvironments where localized oxidation and pigment transformation continue (Zhang et al. 2025; Langston et al. 2021).

**TABLE 2:** Comparative pigment stability and degradation kinetics in selected frozen vegetables at −20°C.

Crop	Pigment	Ea (kJ/mol)	Retention (%)	Kinetics	References
Taro stolon	Chlorophyll a/b	78–72	65	First‐order	Moon et al. (2020)
Lablab bean	β‐carotene/lutein	42–68	70	Zero‐order	Suborna et al. (2024)
Okra	Chlorophyll a/b	82–91	80	First‐order	Wang et al. (2025)
Green beans	Chlorophyll b	106.3	60	First‐order reversible	Zhang et al. (2025), Silva and Sulaiman (2022)
Purple bean	Anthocyanins	56	58	Weibull	Zhang et al. (2023), Shezi et al. (2024)
Broccoli	Chlorophyll + vit C	85–112	82	First‐order	Çalışkan Koç et al. (2025), Pompili et al. (2023)
Spinach	Vit C/chlorophyll	108–115	65	First‐order	Liang et al. (2024); Sui et al. (2023)
Green pea	Chlorophyll/vit C	85–112	68	First‐order	Matabura (2023)
Purple sweet potato	Anthocyanins (pH‐dependent)	26–60	68	First‐order (pH‐dependent)	Palumbo et al. (2022)
Tomato	Lycopene	25–35	45–55	Zero‐order (frozen)	Neri et al. (2020)

Recent studies employing multivariate and spectroscopic approaches (e.g., hyperspectral imaging, PCA, and chemometric modeling) have provided deeper insights into pigment stability in complex food matrices. These approaches enable simultaneous evaluation of multiple quality attributes, revealing strong correlations between pigment degradation, microstructural disruption, and storage conditions. Such integrated analytical frameworks offer improved predictive capability compared to single‐parameter assessments and are particularly valuable for optimizing industrial freezing and storage strategies (Sitoe et al. 2024, 2025).

### 3.1 Chlorophyll Degradation Mechanism

Chlorophylls a and b are the principal pigments responsible for green color and are degraded into pheophytins and pheophorbides under freezing‐induced stress (Wang et al. 2024). The stability and composition of chlorophyll pigments are also influenced by the maturity stage of the vegetable. In immature tissues, chlorophyll concentration is typically high, with chlorophyll a dominating due to active photosynthetic processes. At optimal maturity, a balance between chlorophyll a and b is observed, contributing to stable green coloration. In contrast, overmature tissues exhibit chlorophyll degradation and increased accumulation of degradation products, resulting in reduced green intensity. Chlorophyll a is generally more abundant and directly involved in photosynthesis, while chlorophyll b acts as an accessory pigment and is relatively more stable under stress conditions. During freezing and storage, these differences influence degradation kinetics, with chlorophyll a often degrading faster than chlorophyll b, contributing to progressive color changes. Their stability is critically linked to visual quality, nutritional perception, and consumer acceptance of frozen vegetable products. Under frozen storage and freeze–thaw stress, chlorophyll degradation proceeds through a series of biochemical reactions and physicochemical changes, leading to the loss of green hue and the appearance of yellow–brown tones. The fundamental pathway of chlorophyll breakdown involves enzymatic dechelation, dephytylation, and macrocycle cleavage, which produce colorless or brown catabolites rather than stable green pigments (Tanaka and Ito 2024).

Recent molecular investigations of chlorophyll catabolism highlight a multistep biochemical cascade that initiates with conversion of chlorophyll b to chlorophyll a, followed by removal of central Mg^2+^ and phytol side chains (Tanaka and Ito 2024). Once magnesium is removed by Mg‐dechelatase activity, pheophytins are formed; these are less stable and begin to shift the perceived color toward olive or brown hues. Subsequent actions of enzymes such as chlorophyllase (which hydrolyzes chlorophyll to chlorophyllide plus phytol), pheophorbide a oxygenase (PAO), and red chlorophyll catabolite reductase (RCCR) lead to macrocycle cleavage and formation of phyllobilins, which are colorless degradation products. These reactions have been extensively documented in plant physiology and are central to de‐greening processes in senescence but they also occur under cold‐induced cellular disruption in frozen produce (Wang et al. 2020).

During freezing and subsequent thawing, ice crystal formation and recrystallization cause structural disruption of chloroplasts and vacuoles. This structural damage enhances access of chlorophyll‐degrading enzymes to their substrates, accelerates pH changes, and increases contact with ROS (reactive oxygen species). Low temperature alone does not completely inactivate degradative enzymes in unfrozen water fractions, meaning enzymatic chlorophyll breakdown continues gradually even at −18°C to −20°C typical of commercial frozen storage. Studies on leafy products show that pigment loss during thawing, particularly within 6–12 h of warming, is closely linked to structural compromise of cells rather than intrinsic pigment instability (Chen, Wang, et al. 2024). Cellular acidification, a common consequence of freezing‐induced compartment leakage, promotes pheophytin formation by facilitating Mg^2+^ displacement. Additionally, ROS generated from membrane lipid oxidation can further oxidize chlorophyll derivatives, enhancing the loss of green color. Although antioxidant systems exist in intact tissues, disruption during freezing suppresses these defenses, allowing oxidative degradation to proceed (Zhang et al. 2025). Figure 2 provides a mechanistic overview of pigment degradation pathways, illustrating the transformation of chlorophylls, carotenoids, and anthocyanins under frozen storage conditions.

The figure emphasizes the role of enzymatic activity, oxidative stress, and cellular disruption in driving pigment instability, demonstrating how multiple degradation pathways converge to produce observable color changes.

The rate of chlorophyll degradation under frozen conditions depends on storage temperature stability and thawing dynamics. Lower storage temperatures and minimal temperature fluctuations suppress enzymatic mobility and slow down degradation pathways. Conversely, larger freeze–thaw temperature gradients aggravate structural damage and accelerate pigment breakdown, as observed in experimental leaf vegetable systems where chlorophyll content declines more rapidly with increased thawing temperatures. In tropical vegetable tissues, the interplay between freezing stress, enzymatic activity, and oxidative factors governs the pace of chlorophyll loss. Optimizing pre‐freezing blanching to target residual chlorophyllase and Mgdeche‐latase activities, controlling pH and oxygen exposure, and maintaining stable storage temperatures are critical to mitigating green color loss. In industrial systems, blanching conditions (typically 85°C–98°C for 1–3 min) play a critical role in determining residual chlorophyllase and Mg‐dechelatase activity. Inadequate blanching results in continued enzymatic degradation, while excessive blanching can disrupt cellular integrity and accelerate pigment leaching. Additionally, oxygen availability within packaging systems and water activity in partially frozen matrices significantly influence oxidative degradation pathways. Understanding these mechanistic details provides a basis for predictive modeling and cold chain interventions that preserve visual quality (Labra and Zoffoli 2024; Li et al. 2023).

### 3.2 Carotenoid Oxidation and Isomerization

Carotenoids are lipid‐soluble pigments responsible for the yellow, orange, and red hues in many tropical vegetables (e.g., pumpkin, squash, and peppers) (Meléndez‐Martínez et al. 2023). Unlike chlorophylls, carotenoids are embedded in plastid membranes and lipid matrices, making their stability dependent not only on enzymatic activity but also on physical disruption of cellular structures and oxidative stress during freezing and frozen storage. In frozen tropical vegetables, carotenoid degradation results from oxidative pathways, isomerization, and matrix disruption, which cumulatively lead to diminished color intensity and visual quality (Šeregelj et al. 2022; Meléndez‐Martínez et al. 2023).

#### 3.2.1 Oxidative Degradation and Lipid Interactions

The unsaturated polygenic structure of carotenoids (e.g., β‐carotene and lutein) makes them inherently susceptible to oxidative deterioration. Although low temperatures slow down chemical reactions, the formation of reactive oxygen species (ROS) during freezing and thawing especially when cell membranes are compromised promotes carotenoid oxidation. The ROS attack conjugated double bonds, leading to fragmentation into colorless or less intensely colored compounds and consequent loss of visual quality (Meléndez‐Martínez et al. 2023). In an industrial context, however, some carotenoids demonstrate resilience to freezing stress, likely reflecting protective interactions with lipid environments or structural matrices within the tissues. For example, studies on organic butternut squash ( *Cucurbita moschata* ) subjected to industrial freezing reported that α‐ and β‐carotene bioaccessibility did not significantly decline during the freezing stages, suggesting that certain carotenoid fractions remain structurally intact or retain functional stability under individually quick frozen (IQF) conditions (Kamiloglu et al. 2024).

#### 3.2.2 Impact of Physical Matrix and Cell Structure

Physical disruption of plastids and membranes during freezing leads to the release of carotenoids from their hydrophobic domains into aqueous compartments, a change that can both expose them to oxidative environments and influence their extractability. Carotenoids often associate with lipid bodies or plastoglobules within plant cells; the integrity of these structures plays a key role in pigment stability. In nonfreezing studies of pepper fruits under postharvest conditions, thicker cuticular layers and enhanced lipid exocarp structures were linked with improved carotenoid retention, implying that structural barriers can mitigate oxidative access to carotenoid pools. Although this study did not focus on freezing per se, the mechanistic insight that carotenoid embedding within lipid matrices confers stability is directly relevant to understanding how freezing and freezing‐induced physical disruptions might accelerate pigment loss when these protective barriers are compromised (Holden et al. 2023).

#### 3.2.3 Temperature Effects and Kinetic Behavior

Carotenoid degradation under frozen storage conditions can be described using both zero‐order and first‐order kinetic models, depending on the dominant degradation mechanism and system‐specific conditions. In oxygen‐limited or diffusion‐controlled environments, such as those encountered in tightly packed or low‐permeability systems, carotenoid degradation may follow zero‐order kinetics, where the rate of degradation remains relatively constant and independent of carotenoid concentration. This behavior is typically associated with restricted oxygen diffusion and limited availability of reactive species, which control the overall reaction rate rather than substrate concentration.

In contrast, first‐order kinetics are more representative of systems where oxidative degradation is the primary pathway, particularly in tissues experiencing structural disruption due to freezing and thawing. Under such conditions, the degradation rate becomes proportional to the remaining carotenoid concentration, reflecting progressive oxidative cleavage of conjugated double bonds. As reported by Meléndez‐Martínez et al. (2023), reactive oxygen species generated during membrane damage can initiate carotenoid oxidation, leading to fragmentation into less intensely colored or colorless compounds.

Temperature remains a key factor influencing carotenoid degradation kinetics, even under frozen conditions. Although reaction rates are significantly reduced at subzero temperatures, the presence of unfrozen water fractions allows limited molecular mobility and slow chemical reactions to persist. Experimental findings by Šeregelj et al. (2022) demonstrated that carotenoids in low‐moisture or freeze‐dried systems can exhibit zero‐order degradation behavior, with increasing storage temperature accelerating degradation rates and reducing half‐life values. Furthermore, temperature fluctuations during frozen storage can enhance both oxidative reactions and trans–cis isomerization, contributing to reduced chromophoric stability and color intensity.

The physical structure of the food matrix also plays a critical role in determining degradation kinetics. Carotenoids embedded within intact plastid membranes or lipid matrices exhibit enhanced stability due to limited exposure to oxygen and prooxidant species. However, freezing‐induced structural disruption increases carotenoid accessibility to oxidative environments, potentially shifting degradation behavior toward first‐order kinetics. In industrial freezing scenarios, such as individually quick frozen (IQF) systems, studies by Kamiloglu et al. (2024) indicate that certain carotenoid fractions may retain structural integrity and bioaccessibility despite freezing stress, suggesting that matrix protection can modulate degradation pathways.

Overall, carotenoid degradation in frozen tropical vegetables is governed by a combination of oxidative mechanisms, temperature effects, and structural changes within the food matrix. The coexistence of multiple degradation pathways may lead to deviations from simple kinetic models, highlighting the importance of selecting appropriate model systems and considering both chemical and physical factors when predicting carotenoid stability during frozen storage.

#### 3.2.4 Influence of Bioaccessibility and Processing Context

Beyond absolute content, bioaccessibility the fraction of carotenoid that becomes available for digestion is also affected by freezing and processing. Bioaccessibility, defined as the fraction of a compound released from the food matrix during digestion and available for absorption, is also influenced by freezing‐induced structural changes. In industry‐scale IQF processes, butternut squash maintained carotenoid bioaccessibility through freezing stages, suggesting that while chemical integrity may remain relatively stable, the spatial redistribution of carotenoids within tissue matrices can influence their functional availability (Kamiloglu et al. 2024). Preservation of bioaccessibility despite freezing suggests that carotenoid compounds can remain intact within the lipid and cellular matrix even when structural changes occur, but this may vary significantly with vegetable type and freezing conditions.

### 3.3 Anthocyanin Stability

#### 3.3.1 Anthocyanin Breakdown and Polymerization

Anthocyanins are hydrophilic flavonoid pigments largely responsible for red–purple coloration in certain tropical vegetable tissues and pigmented cultivars. Their stability in frozen matrices is governed by chemical form (glycosylation/acylation), pH, copigmentation, interaction with macromolecules (e.g., pectin), oxidative environment, and physical stress from freeze–thaw cycles (Xue et al. 2024; Tkaczyńska et al. 2024). Compared with chlorophylls and carotenoids, anthocyanins are particularly sensitive to changes in the aqueous microenvironment; therefore, freeze‐induced disruption of vacuoles and the resulting shifts in pH and ionic strength strongly influence anthocyanin color expression and degradation (Xue et al. 2024).

#### 3.3.2 Chemical Transformations

Under freezing and especially during thawing, anthocyanin molecules can undergo hydrolytic cleavage of glycosidic bonds, oxidation of the flavylium core, and condensation/polymerization reactions that convert monomeric, brightly colored anthocyanins into brownish, high‐molecular‐weight pigments (polymeric anthocyanins and flavanol–anthocyanin adducts). Hydrolysis is promoted by increased exposure of anthocyanins to acidic or enzymatic conditions when vacuolar integrity is lost; oxidation and polymerization are favored in the presence of reactive oxygen species produced by lipid peroxidation and metal‐catalyzed reactions during membrane rupture (Tkaczyńska et al. 2024). These pathways reduce monomeric anthocyanin concentration and shift spectral properties toward longer wavelengths and lower chroma.

#### 3.3.3 pH, Molecular Form and Color Expression

Anthocyanins interconvert between several forms depending on pH (flavylium cation, quinoidal base, carbinol pseudobase, chalcone), and small pH changes caused by cellular leakage during freezing can dramatically alter apparent color (e.g., vivid red → purple → brown). Studies focused on extraction/stability show that lower pH favors the flavylium form (more red), whereas neutral to alkaline microenvironments favor colorless or brown derivatives; thus, freeze‐induced acid–base shifts are central to color loss in frozen tissues (Gamage and Choo 2023). This pH sensitivity also explains why slight post‐processing acidification or buffering can substantially improve anthocyanin color retention.

#### 3.3.4 Role of Copigmentation and Macromolecular Interactions

Anthocyanin color stability is enhanced by copigmentation (complex formation with other phenolics) and by binding to macromolecules such as pectin and proteins. Importantly for frozen vegetables, pectin released from damaged cell walls can form non‐covalent complexes with anthocyanins that reduce their chemical reactivity and protect against oxidation and polymerization; recent mechanistic reviews demonstrate that pectin–anthocyanin complexes increase thermal and storage stability and can improve freeze–thaw resilience of anthocyanin color (Shi, Fu, et al. 2024; Shi, Guo, et al. 2024). This explains empirical observations where tissues with higher pectin content or controlled pectin structure show better anthocyanin retention after freezing.

#### 3.3.5 Freeze–Thaw Cycles, Physical Stress and Kinetics

Repeated freeze–thaw cycles accelerate anthocyanin loss by repeatedly disrupting vacuolar sequestration, increasing exposure to enzymes (e.g., oxidases) and oxygen, and enhancing molecular mobility during partial thaw phases that permit reaction kinetics to proceed. Recent experimental work on freeze–thaw pretreatments found that such cycles change anthocyanin kinetics and promote formation of polymeric pigments and decreased monomeric anthocyanin recovery effects that are mitigated by minimizing temperature fluctuations or applying protective pretreatments (Llavata et al. 2024). These findings indicate that cold‐chain stability not just the nominal storage temperature critically determines anthocyanin retention.

#### 3.3.6 Stabilization Strategies: Encapsulation, Copigmentation, and Formulation

Because anthocyanins are inherently labile, multiple stabilization strategies that are applicable to frozen foods have been developed and tested recently. Encapsulation systems typically involve biopolymeric carriers such as polysaccharides (e.g., maltodextrin, gum arabic, pectin), proteins (e.g., whey protein isolate), or lipid‐based systems (e.g., nanoemulsions and liposomes), which act as protective matrices to limit oxygen exposure and stabilize anthocyanins during freeze–thaw stress (Islam et al. 2026). Encapsulation (micro‐/nano‐carriers, double emulsions, or polymer matrices) can protect anthocyanins from oxygen and enzymatic attack during freeze–thaw stress; a 2024 review summarizes polymer‐based encapsulation approaches that significantly improve anthocyanin retention under thermal and freeze–thaw challenges (Rosales‐Murillo et al. 2024). In practice, co‐formulation with pectin, the use of copigments, or controlled acidification prior to freezing are pragmatic interventions to limit monomer loss and polymer formation in frozen vegetable products (Rahman et al. 2026).

#### 3.3.7 Practical Implications for Tropical Vegetables

For pigmented tropical vegetables, maintaining anthocyanin color during frozen storage requires: (1) minimizing freeze–thaw events and temperature excursions in the cold chain, (2) optimizing pre‐freezing handling to limit vacuolar rupture (gentle blanching, rapid cooling), and (3) considering formulation strategies such as mild acidification, pectin retention, or encapsulation where processing permits. Integrating mechanistic knowledge of pH‐dependent transformations, copigmentation, and polymer formation into kinetic models will improve shelf life predictions for anthocyanin color in frozen tropical vegetables. Table 2 compiles reported data on pigment stability in various tropical vegetables under frozen storage, including degradation trends, influencing factors, and experimental conditions.

## 4 Texture Degradation

Texture degradation is one of the most significant quality losses in frozen tropical vegetables during extended storage and is primarily governed by the integrity of cell walls, plasma membranes, turgor pressure, and intercellular adhesion (Billah, Zannat, et al. 2025). Unlike fresh tissues, frozen vegetables experience a combination of physical stress induced by ice formation and recrystallization, biochemical softening driven by residual cell‐wall–modifying enzymes, and structural failure of membranes and middle lamellae, all of which act synergistically rather than independently. From a mechanistic perspective, texture deterioration follows a hierarchical sequence: initial ice crystal formation disrupts cellular compartmentalization, resulting in loss of turgor pressure and membrane permeability; this physical damage subsequently enhances enzyme–substrate interactions, accelerating pectin solubilization and depolymerization; finally, prolonged frozen storage promotes irreversible cell wall collapse and weakened cell‐to‐cell adhesion, leading to soft, mushy textures upon thawing. Tropical vegetables, characterized by high moisture content and relatively thin cell walls, are particularly vulnerable to these degradation pathways. These structural changes are commonly quantified using instrumental techniques such as Texture Profile Analysis (TPA), which measures parameters including hardness, cohesiveness, springiness, and chewiness, as well as shear force and modulus of elasticity. Reductions in hardness (20%–60%) and shear force have been strongly correlated with microstructural damage observed through microscopy, providing quantitative validation of texture degradation mechanisms.

### 4.1 Ice Crystal Formation and Recrystallization

Ice crystal formation is the primary physical driver of texture degradation in frozen tropical vegetables and initiates a cascade of structural and biochemical changes that culminate in softening. During freezing, water in the intercellular spaces nucleates first due to lower solute concentration, forming extracellular ice crystals. This process increases the osmotic pressure of the unfrozen intracellular phase, promoting water efflux from the cytoplasm and resulting in cellular dehydration and volume shrinkage (Rahman et al. 2022). The loss of intracellular water directly reduces turgor pressure, which is a major contributor to firmness and crispness in plant tissues.

The rate of freezing plays a decisive role in determining ice crystal size and distribution. Under slow freezing conditions (e.g., conventional static freezers operating near −18°C to −20°C), water molecules have sufficient time to migrate and aggregate into fewer but larger ice crystals. These large crystals exert significant mechanical stress on the cell wall–membrane complex, leading to membrane puncture, cell wall distortion, and irreversible loss of structural integrity. In contrast, rapid freezing techniques, such as blast freezing at −40°C or cryogenic freezing, induce rapid nucleation and limit crystal growth, resulting in a fine ice crystal network that causes comparatively less mechanical damage to cellular architecture (Silva and Sulaiman 2022).

During frozen storage, ice recrystallization represents a critical secondary mechanism of texture deterioration. Recrystallization occurs when small ice crystals partially melt and refreeze into larger crystals under conditions of temperature fluctuation, even within narrow ranges commonly encountered in commercial cold chains. This phenomenon progressively amplifies mechanical damage by repeatedly stressing cell walls and membranes, thereby exacerbating cell rupture and loss of cohesion between adjacent cells. Recrystallization is particularly detrimental in tropical vegetables due to their high water content and relatively thin primary cell walls, which offer limited resistance to repeated mechanical stress.

Empirical evidence demonstrates a strong association between ice recrystallization phenomena during frozen storage and textural degradation in plant‐based foods. Ice recrystallization the growth of larger ice crystals at the expense of smaller ones during storage, especially under temperature fluctuations is widely recognized as a key driver of microstructural damage and texture softening due to cellular membrane rupture and moisture migration, which reduce hardness, chewiness, and cutting force in frozen food materials. Research on frozen foods has identified that larger and redistributed ice crystals formed during prolonged storage lead to deterioration of structural integrity and increased textural losses compared with samples exhibiting finer ice morphology, confirming the role of recrystallization in quality decline (Sun et al. 2023; Roos 2021). Similar trends have been observed in other high‐moisture vegetables, where repeated freeze–thaw events significantly accelerate tissue collapse and drip loss upon thawing.

At the microstructural level, ice crystal growth disrupts the continuity of the plasma membrane, increasing permeability and eliminating selective transport. This loss of compartmentalization allows intracellular solutes, enzymes, and substrates to mix, thereby intensifying secondary biochemical degradation pathways, including pectin depolymerization and cell wall loosening. Furthermore, moisture migration from the cytoplasm to the extracellular space during freezing leads to localized concentration of solutes, which alters ionic strength and promotes destabilization of cell wall polysaccharide networks (Takahashi et al. 2018).

From a kinetic standpoint, the extent of ice crystal damage is not solely a function of storage temperature but is highly sensitive to thermal history, including cooling rate, temperature oscillation amplitude, and storage duration. Vegetables subjected to stable, low‐temperature storage with minimal fluctuation show significantly better texture retention than those exposed to repeated thermal abuse, even when the average temperature remains unchanged. These findings underscore the importance of cold‐chain stability in preserving textural quality in frozen tropical vegetables (Ullah et al. 2014). Overall, ice crystal formation and recrystallization serve as the initiating events that drive subsequent enzymatic and structural degradation. Effective mitigation strategies such as rapid freezing, temperature‐stable storage, and the use of cryoprotective treatments are therefore essential for maintaining the texture integrity of frozen tropical vegetables throughout long‐term storage.

### 4.2 Enzymatic Softening (PME, PG, β‐Galactosidase)

Enzymatic softening is a critical biochemical contributor to texture degradation in frozen tropical vegetables and typically acts as a secondary mechanism following physical damage induced by ice crystal formation. Although blanching is widely applied prior to freezing to inactivate endogenous enzymes, complete inactivation is rarely achieved under industrial conditions. As a result, residual activities of pectin methyl esterase (PME), polygalacturonase (PG), and β‐galactosidase persist during frozen storage and contribute to gradual cell wall weakening (Wang et al. 2025). PME catalyzes the demethylation of homogalacturonan regions of pectin, generating free carboxyl groups that increase the susceptibility of pectin chains to depolymerization. While limited PME activity can promote calcium‐mediated cross‐linking and transient firmness enhancement, excessive or prolonged demethylation during frozen storage facilitates calcium dissociation and pectin solubilization, ultimately leading to loss of intercellular adhesion. PG further accelerates this process by cleaving α‐(1 → 4)‐linked galacturonic acid residues, reducing the molecular weight of pectin polymers and promoting middle lamella disassembly (Wang et al. 2024).

β‐Galactosidase contributes to softening by hydrolyzing galactosyl side chains of rhamnogalacturonan I, thereby destabilizing the pectin network and increasing the accessibility of the backbone to other pectinolytic enzymes. The combined action of these enzymes leads to a shift from insoluble, covalently bound pectin to water‐soluble fractions, which is closely associated with textural softening in frozen vegetables. Residual enzymatic activity has been reported to persist for several months during frozen storage, particularly in tropical vegetables with high moisture content. Mondal et al. (2022) observed measurable PME activity in lablab bean and taro stolon tissues after up to 6 months of storage at −20°C. Importantly, ice recrystallization during storage releases unfrozen water into localized microenvironments, enhancing enzyme mobility and enabling limited enzymatic reactions even under subzero conditions. Although reaction rates are significantly reduced, the cumulative effect over extended storage periods becomes technologically relevant. Overall, enzymatic softening complements physical damage caused by ice crystals by accelerating pectin degradation once cellular compartmentalization is compromised. Effective control of enzymatic softening therefore requires not only optimized blanching protocols but also stable low‐temperature storage to minimize ice recrystallization and the formation of enzyme‐active microdomains.

### 4.3 Cell Wall and Membrane Structural Damage

Structural damage to cell walls and cellular membranes represents the final and largely irreversible stage of texture degradation in frozen tropical vegetables. Once physical disruption by ice crystal formation and biochemical weakening by residual enzymatic activity have progressed, the plant tissue loses its ability to recover structural integrity upon thawing. The cell wall–membrane complex, which normally maintains cellular shape and mechanical strength, becomes increasingly compromised during prolonged frozen storage (Giannakourou et al. 2020).

Microscopic investigations, particularly using scanning electron microscopy (SEM) and confocal laser scanning microscopy (CLSM), consistently reveal pronounced microstructural alterations in frozen tropical vegetables. These include collapsed and distorted cell walls, ruptured plasma membranes, enlarged intercellular spaces, and separation of adjacent cells, especially after 9–12 months of storage at approximately −20°C (Zhu et al. 2020). Such damage reflects the combined effects of mechanical stress from ice crystals, osmotic dehydration, and enzymatic depolymerization of cell wall polysaccharides.

Loss of membrane integrity eliminates selective permeability and prevents the reestablishment of intracellular turgor during thawing. As a consequence, water released during ice melting is unable to be reabsorbed by damaged cells, resulting in drip loss, tissue flaccidity, and reduced cohesiveness. This phenomenon explains why frozen–thawed vegetables often exhibit acceptable appearance in the frozen state but suffer severe textural collapse after cooking or reheating.

From a mechanical standpoint, structural failure of the middle lamella rich in calcium‐bridged pectin is particularly detrimental. Disruption of these intercellular junctions leads to cell separation rather than cell rupture, producing a soft and mealy texture. Instrumental texture measurements show significant reductions in hardness, chewiness, and resilience that correlate strongly with observed microstructural damage. These changes typically follow first‐order or Weibull kinetic models, with reported softening rate constants (*k*) ranging from 0.015 to 0.035 month^−1^, depending on vegetable species, blanching intensity, and storage conditions (Wang et al. 2024).

Moisture migration further exacerbates structural damage. During freezing, intracellular water migrates toward extracellular ice crystals, leading to localized dehydration of the cytoplasm and concentration of solutes, which weakens hydrogen bonding and disrupts polysaccharide–polysaccharide interactions within the cell wall matrix. Repeated recrystallization during storage intensifies this effect, progressively destabilizing cellulose–hemicellulose–pectin networks and reducing the resistance of tissues to mechanical stress.

Overall, cell wall and membrane structural damage represents the cumulative outcome of physical, biochemical, and thermodynamic stresses experienced during freezing and frozen storage. Once this level of degradation is reached, textural quality cannot be fully restored, highlighting the importance of preventive strategies such as rapid freezing, optimized blanching, and strict temperature control to preserve the microstructural integrity of frozen tropical vegetables.

## 5 Kinetic Modeling and Prediction of Quality Degradation

Modeling approaches for predicting quality degradation in frozen tropical vegetables can be broadly categorized into three groups: (i) mechanistic models, which are based on physical and biochemical principles such as heat and mass transfer and enzyme kinetics; (ii) empirical or kinetic models, including zero‐order, first‐order, and Weibull models, which describe degradation behavior using experimental data; and (iii) data‐driven models, such as artificial neural networks and machine learning algorithms, which capture complex nonlinear relationships without explicit mechanistic assumptions. This classification provides a clearer framework for selecting appropriate modeling strategies based on data availability and application requirements (Table 3).

**TABLE 3:** Modeling approaches for predicting color and texture degradation in frozen or low‐temperature stored fruits and vegetables.

Model type	Dependent variables	Advantages	Limitations	References
First‐order; first‐order reversible; Arrhenius	Chlorophyll a, b; Hunter a*, b*; total color difference (frozen green beans)	Simple; good fit up to 250 days; enables Ea estimation and temperature extrapolation; reversible form captures color plateau	Validated only for green beans; irreversible first‐order alone is inadequate once equilibrium is reached	Martins and Silva (2002)
First‐order; Arrhenius (variable storage)	Vitamin C in frozen green vegetables	Describes ascorbic acid loss under constant and variable frozen storage; allows prediction for different temperature scenarios	Only vitamin C modeled; no texture or detailed color; limited temperature and product range	Giannakourou and Taoukis (2003)
First‐order; fractional‐conversion; Arrhenius	Texture (firmness); vitamin C; reducing sugars; starch (frozen green beans)	Fractional‐conversion improves texture fit; first‐order adequate for vitamin C and starch; Ea values obtained for multiple quality indices	Developed for green beans; first‐order not suitable for texture; three storage temperatures only	Martins and Silva (2003)
Fractional‐conversion; Arrhenius (isothermal and non‐isothermal)	Firmness; CIE L*, a*, b*; vitamin C (pumpkin)	Single framework for color, texture and vitamin C; applicable to constant and fluctuating frozen temperatures	Some fits show moderate R^2^, especially for color under non‐isothermal storage; parameters product‐ and profile‐specific	Gonçalves et al. (2011)
Zero‐order; first‐order; Arrhenius	Individual carotenoids (lycopene, β‐carotene, α‐carotene, lutein) in frozen carrot slices	Allows separate kinetic description per carotenoid; provides k and Ea for long‐term frozen storage; supports process design for color stability	Results specific to a lycopene‐rich carrot cultivar; kinetic behavior differs between carotenoids; not directly generalizable	Behsnilian and Mayer‐Miebach (2017)
Weibull‐type model	Total anthocyanins; color of purple sweet‐potato anthocyanin colorant	Captures nonlinear, biphasic pigment loss; in some cases fits better than first‐order; parameters enable comparison of stability conditions	Applied to extracted colorant, not whole tissue; conditions ambient/refrigerated, not frozen; system‐specific	Quan et al. (2019)
Williams–Landel–Ferry (WLF) equation	Nonenzymatic browning rate constants in carbohydrate/vegetable model systems	Describes combined moisture–temperature effects; links browning rate to glass transition temperature	Mainly tested on model and freeze‐dried systems; requires Tg data; application to complex foods is nontrivial	Pilar and Karel (1993)

To overcome these limitations, advanced data‐driven and multivariate modeling frameworks, including artificial neural networks (ANNs), response surface methodology (RSM), and genetic algorithms, have gained prominence due to their ability to capture nonlinear interactions among temperature history, moisture migration, enzyme activity, oxygen exposure, and microstructural damage. Recent studies demonstrate that such hybrid and machine‐learning‐based models significantly outperform traditional kinetics in predicting quality trajectories, particularly when multiple degradation mechanisms operate simultaneously during long‐term frozen storage. Consequently, integrating mechanistic kinetic models with advanced computational tools is increasingly viewed as a robust strategy for improving predictive accuracy, optimizing processing parameters, and enabling risk‐based decision‐making in frozen tropical vegetable preservation (Du et al. 2022; Yang et al. 2023). Table 3 presents a comparative overview of different modeling approaches applied to predict quality degradation in frozen vegetables, including kinetic, statistical, and machine learning models.

### 5.1 Classical Kinetic Models

Classical kinetic models remain the backbone for quantifying and comparing rates of quality loss (color, texture, nutrients) in frozen vegetables because they provide simple, interpretable parameters (rate constants, half‐lives) that can be used for shelf life prediction and process optimization. The most commonly applied formulations are first‐order, zero‐order, and Weibull models. Choosing the correct model depends on the underlying degradation mechanism (e.g., enzyme‐limited vs. diffusion‐limited vs. multiphase reactions) and on goodness‐of‐fit to empirical data (Cui et al. 2023).

#### 5.1.1 First‐Order Kinetics

First‐order kinetics assume the instantaneous rate of loss is proportional to the remaining amount of the quality attribute:

(1) $$\frac{\mathrm{dC}}{\mathrm{dt}}=-\mathrm{kC}$$

which integrates to $C_{t}=C_{0}e^{-\mathrm{kt}}$. First‐order behavior is frequently observed for pigment decay (e.g., chlorophyll) and firmness loss when a single dominant degradation pathway (or an enzyme with simple inactivation kinetics) controls the process. First‐order models give directly interpretable metrics such as the rate constant (*k*) and half‐life $t_{1/2}=\ln2/k$, which are convenient for comparing species, treatments, or storage temperatures. Recent reviews and experimental work confirm that many vegetable quality indices under isothermal frozen storage are well described by first‐order kinetics, making it a practical first choice in modeling workflows (Liao et al. 2024).

#### 5.1.2 Zero‐Order Kinetics

Zero‐order kinetics describe situations where the absolute rate of change is approximately constant and independent of the remaining concentration:

(2) $$C_{t}=C_{0}-\mathrm{kt}$$

Zero‐order behavior can arise when the reaction rate is limited by an external factor (e.g., oxygen diffusion through packaging, limited enzyme availability, or surface‐limited reactions) rather than by the pool of reactant itself. For example, pigment oxidation in oxygen‐restricted packages or cases where mass transfer limits pigment conversion can follow zero‐order trends. Use zero‐order models when empirical plots of C vs. t are linear and when residuals indicate poor fit with exponential (first‐order) models (Liao et al. 2024).

#### 5.1.3 Weibull Model

The Weibull expression provides flexibility to capture non‐exponential degradation profiles that arise from multiphase or heterogeneous processes:
(3)

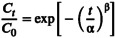

Here α is a time‐scale parameter and β (shape parameter) controls whether the hazard (instantaneous rate) increases (β > 1), decreases (β < 1), or is constant (β = 1 → first‐order). The Weibull model is especially useful when degradation shows an initial lag, a shoulder, or tail behaviors commonly observed when physical damage (e.g., ice crystal rupture) and biochemical reactions (residual enzyme activity) act together. Recent methodological work on the Weibull distribution emphasizes its interpretability and superior fit for many complex food‐degradation data sets and provides guidance on parameter interpretation and regression approaches for experimental shelf life studies (Gómez et al. 2024).

### 5.2 Temperature Dependence and Arrhenius Modeling

Temperature critically influences the rates of quality degradation in frozen tropical vegetables, affecting both physical phenomena (e.g., ice recrystallization) and biochemical reactions (e.g., enzyme activity, pigment oxidation). Classical kinetic parameters such as rate constants (*k*) are temperature‐dependent and can be predicted across storage conditions using Arrhenius‐type models, which relate reaction rates to thermal energy and activation barriers.

#### 5.2.1 Arrhenius Equation

The Arrhenius relationship expresses how the rate constant (*k*) varies with absolute temperature (T) as follows:

(4) $$k=k_{0}\exp\;\left(-\frac{E_{a}}{\mathrm{RT}}\right)$$

where *k*_0_ is the pre‐exponential factor (frequency of collisions with appropriate orientation), E_a_ is the activation energy (kJ·mol^−1^), R is the universal gas constant (8.314 J·mol^−1^·K^−1^), and T is absolute temperature (*K*). Activation energy represents the sensitivity of a degradation process to temperature: high E_a_ indicates greater acceleration of quality loss with temperature rise, while low E_a_ suggests relative temperature insensitivity.

Arrhenius modeling provides a quantitative basis for predicting quality loss under changing temperature scenarios from stable deep‐freezing to dynamic cold‐chain excursions enabling the extrapolation of short‐term accelerated tests to long‐term storage behavior. Several recent studies demonstrate the utility of Arrhenius models in frozen food contexts. In research on frozen peas and kale, chlorophyll degradation over a range of subzero temperatures was well described by Arrhenius plots, with estimated activation energies indicating a significant dependence of pigment loss on temperature fluctuations during storage (Martins and Silva 2002). For texture attributes such as firmness and cutting force in frozen leafy vegetables, Arrhenius models have successfully linked temperature history profiles with rate constants, confirming that even small increases from −20°C to −10°C can accelerate softening rates disproportionately due to enhanced ice recrystallization kinetics and enzyme mobility in unfrozen water fractions (Sun et al. 2023).

#### 5.2.2 Modeling Under Non‐Isothermal Conditions

Traditional Arrhenius analysis assumes isothermal conditions, but real cold chains are frequently dynamic. Recent modeling frameworks extend Arrhenius concepts to non‐isothermal time–temperature profiles, integrating small temperature fluctuations over time to compute an effective degradation integral:

(5) $$\int_{0}^{t}k\left(T\left(t^{\prime }\right)\right)\;dt^{\prime }$$

This approach known as the time–temperature superposition or cumulative Arrhenius integration allows realistic prediction of quality loss under variable storage profiles. It has been applied to freezing and cold‐chain modeling of produce, demonstrating improved predictive accuracy compared to static isothermal fits. These developments highlight how Arrhenius modeling remains foundational but must be adapted to practical storage realities in the frozen vegetable supply chain (Tsironi et al. 2020).

#### 5.2.3 Integration With Shelf Life Prediction and Risk Assessment

Once kinetic parameters such as activation energy (E_a_) and pre‐exponential factors are experimentally determined for specific quality attributes, such as chlorophyll retention, total color difference (ΔE), or firmness measured by texture profile analysis (TPA), Arrhenius‐based models become powerful tools for predicting quality evolution during frozen storage. These models enable quantitative shelf life projection under defined thermal histories, including the application of accelerated storage tests that allow rapid estimation of long‐term quality stability. Importantly, Arrhenius kinetics facilitate comparative evaluation of alternative storage and distribution scenarios, supporting risk assessment associated with temperature abuse, non‐isothermal conditions, and cold‐chain interruptions. When coupled with empirical degradation models, such as first‐order or Weibull kinetics, Arrhenius relationships allow the generation of time–temperature quality surfaces, offering a dynamic and mechanistic description of quality trajectories rather than static end point assessments. Consequently, Arrhenius modeling bridges fundamental understanding of temperature‐dependent degradation mechanisms with practical decision‐making frameworks for optimizing freezing protocols, cold‐chain design, and quality assurance strategies in frozen tropical vegetable systems (Mohd Ali et al. 2022).

### 5.3 Response Surface Methodology and Multiparameter Optimization

Response Surface Methodology (RSM) is an advanced statistical design and optimization framework that enables the simultaneous analysis of multiple process and storage variables influencing quality degradation in frozen tropical vegetables. Unlike simple one‐factor‐at‐a‐time approaches, RSM constructs empirical surfaces that approximate the true response function of quality attributes (color, texture, nutritional retention, enzyme activity) across a multidimensional factor space (Joudi‐Sarighayeh et al. 2023). This allows researchers and industry practitioners to identify optimal processing windows, understand interaction effects, and minimize deleterious outcomes such as pigment loss, softening, and nutrient degradation. Given the complex interplay between pre‐processing parameters (e.g., blanching time/temperature) and frozen storage conditions (e.g., cooling rate, packaging atmosphere, storage temperature), RSM is particularly suited for the integrated optimization needed in frozen tropical vegetable preservation (Stavropoulou et al. 2022).

#### 5.3.1 Theoretical Basis and Model Formulation

RSM begins with the selection of a suitable experimental design (e.g., central composite design, Box–Behnken design) to systematically vary key independent factors around a nominal center point. A second‐order polynomial model is then fitted to the resulting data:
(6)

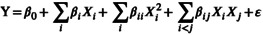

where Y is the response (e.g., color retention, firmness), X_i are independent variables (e.g., blanching temperature, freezing rate, storage time), β coefficients represent system sensitivities, and ε is the error term. The response surface and contour plots then guide interpretation of main effects and interactions. This multivariate modeling is critical when variables interact nonlinearly; for example, blanching temperature can influence both enzyme inactivation and physical tissue strength, with consequences for subsequent freezing and storage dynamics (Sharma et al. 2025).

#### 5.3.2 Applications to Blanching Optimization

Blanching is a vital pre‐freezing step for most vegetables to mitigate the activity of peroxidase (POD), polyphenol oxidase (PPO), and other degradative enzymes. However, excessive blanching can weaken cell walls, leach water‐soluble nutrients (e.g., ascorbic acid), and increase susceptibility to texture loss during freezing. Recent studies applying Response Surface Methodology (RSM) have demonstrated its effectiveness for optimizing blanching conditions in various vegetables to maximize quality retention while minimizing enzymatic activity. For example, in spinach, RSM identified that a blanching temperature of approximately 98°C for 2–3 min provided optimal retention of color and texture, while effectively reducing enzyme activity, highlighting the balance between thermal inactivation of peroxidase and preservation of tissue integrity (Lim et al. 2024). Similarly, in sword bean (*Canavalia gladiate*), RSM optimization of blanching temperature and time significantly reduced peroxidase activity and improved quality outcomes, with the statistical models allowing precise prediction of quality attributes based on process variables (Zhou et al. 2012).

In leafy vegetables such as water spinach, RSM combined with drying experiments after blanching showed that blanching parameters strongly influenced nutritional quality, including moisture, vitamin C, and β‐carotene retention. Optimized blanching conditions identified through the RSM model maintained higher nutrient levels and improved textural properties compared to non‐optimized treatments (Billah, Rahman, et al. 2025).

#### 5.3.3 Optimization of Freezing and Storage Conditions

Beyond pre‐processing, RSM is increasingly used to refine freezing parameters and storage environments that influence ice morphology, recrystallization, and subsequent quality stability. RSM has been applied to plant tissues to compare cryogenic immersion (e.g., liquid nitrogen) with conventional air freezing, revealing that interaction effects (e.g., freezing rate × tissue thickness) significantly influence ice crystal size distribution and texture outcomes. Numerical simulation studies of freezing and thawing processes have used RSM to fit predictive models that relate freezing time and thawing time to variables such as block size, salt content, and operating conditions. By fitting second‐order polynomial response surfaces to simulation data, researchers were able to rapidly estimate how different freezing conditions affect process duration and thereby inform optimization of freezing protocols for quality preservation. These RSM models highlighted significant interactive effects of sample geometry and environmental conditions on freezing dynamics, supporting realistic optimization of cold‐chain practices (Golzarijalal et al. 2023). Recent bibliometric analysis shows that RSM is increasingly applied to optimize storage quality and shelf life responses for fruits and vegetables by modeling interactions among storage temperature, time, packaging, and pretreatments. The thematic analysis from a 2025 study found “optimization,” “storage,” and “shelf life” as major trending RSM research topics in postharvest technology, indicating broad adoption of RSM for modeling complex storage conditions alongside processing variable (Sivaji et al. 2025).

#### 5.3.4 Key Quality Responses

The effectiveness of Response Surface Methodology in frozen vegetable research depends strongly on the selection of sensitive and meaningful response variables that accurately reflect quality degradation pathways. Among these, color attributes such as L* (lightness), a* (greenness–redness), b* (blueness–yellowness), chroma, and total color difference (ΔE) are most frequently employed to quantify pigment stability and visual acceptability during frozen storage. These parameters are typically measured using instrumental colorimetry or, increasingly, hyperspectral and imaging‐based systems, which allow spatially resolved assessment of discoloration (Draghici et al. 2023). Textural responses are equally critical, as consumer rejection of frozen vegetables is often driven more by softening than by color loss. Instrumental texture profile analysis (TPA) parameters such as hardness, cohesiveness, chewiness, springiness, and shear force are commonly incorporated into RSM models to capture mechanical integrity after freezing and thawing (Paciulli et al. 2014). In addition, nutritional markers, including ascorbic acid retention, carotenoid content, and antioxidant capacity, are frequently integrated as secondary or co‐optimized responses to ensure that process optimization does not compromise nutritional quality (Pérez‐Calderón et al. 2019).

Biochemical indicators, particularly residual enzyme activities (peroxidase, polyphenol oxidase, pectin methylesterase), serve as mechanistic proxies for long‐term quality stability and are often included to link empirical optimization with underlying degradation pathways. Finally, drip loss and moisture retention are used as indirect indicators of cellular integrity and membrane damage following thawing. By simultaneously modeling these responses, RSM enables the identification of processing and storage conditions that maximize overall quality rather than optimizing individual attributes in isolation (Suwan 2015; Zhang et al. 2024).

### 5.4 Artificial Neural Networks (ANNs) and Machine Learning

Artificial Neural Networks (ANNs) and broader machine learning (ML) techniques have emerged as powerful tools for modeling complex, nonlinear quality degradation phenomena in food systems, including frozen produce. Unlike classical kinetic models, which often assume simple reaction orders and static conditions, ANN and ML models can learn underlying patterns from large datasets encompassing multiple interacting factors (e.g., temperature history, physicochemical attributes, sensor signals), making them particularly valuable for predicting dynamic quality changes such as color fading and texture softening under real‐world cold‐chain conditions (Siddique et al. 2025). A recent comprehensive review highlights the breadth of ML applications in fruit and vegetable storage, noting that these models can capture nonlinear relationships among storage conditions, physicochemical properties, and quality outcomes (e.g., firmness, pigment retention) without requiring explicit mechanistic formulations. ML models can be continuously refined with new data, improving robustness and predictive accuracy over time, illustrating the adaptability of data‐driven approaches in complex quality prediction tasks (Wang et al. 2024).

#### 5.4.1 Machine Learning Models and Their Predictive Capabilities

ANNs, particularly feedforward backpropagation networks, remain among the most commonly applied architectures for shelf life and quality prediction due to their capacity to approximate nonlinear mappings between input features and output responses. These models have been demonstrated to yield high predictive accuracy with low error metrics when trained on quality indices and storage conditions across diverse food matrices. For instance, ANN‐based shelf life prediction models for fresh produce incorporation of physicochemical parameters such as color, firmness, moisture content, and storage time have shown low relative error and high correlation coefficients, confirming their utility in food quality prediction (Sarkar et al. 2023).

Support vector regression (SVR), random forests, and gradient boosting algorithms represent alternative ML approaches that have also exhibited strong performance in shelf life prediction tasks. In a recent study on potato storage with evaporative cooling, ensemble models such as XGBoost achieved high accuracy in predicting shelf life based on environmental and quality data, demonstrating the broader potential of tree‐based ML models beyond neural architectures.

More advanced deep learning models including convolutional neural networks (CNNs) and recurrent neural networks (RNNs) are increasingly used for real‐time prediction and temporal modeling. CNNs, for example, have been applied to shelf life prediction of fresh produce by analyzing temperature simulation data to forecast quality endpoints as a function of thermal history. This approach underscores the value of deep architectures in handling time‐dependent, multivariate input sequences for dynamic storage scenarios (Kongwong et al. 2021).

#### 5.4.2 Integration With Nondestructive Sensing and Cold‐Chain Data

One of the key strengths of ANN and ML models lies in their ability to integrate heterogeneous data sources including sensor outputs (temperature, humidity), spectroscopy (e.g., near‐infrared), imaging features, and biochemical indices into a unified predictive framework. ML models can thus provide real‐time or near‐real‐time predictions of quality attributes without destructive sampling, enabling proactive quality management within cold chains. For example, modern approaches combine ML with nondestructive spectroscopic measurements to monitor changes in pigment intensity and cell structure, linking spectral features directly to quality metrics. While these approaches are still emerging in the context of frozen vegetables specifically, the demonstrated success in other produce systems suggests clear applicability when tailored to the unique degradation pathways of tropical vegetables (Chen, Fan, et al. 2024; Pandey et al. 2023).

#### 5.4.3 Challenges, Interpretability, and Model Reliability

Despite their predictive power, ANN and ML models are not without limitations. ML models can suffer from overfitting, data bias, and decreased generalizability when trained on limited or poorly representative datasets. Comprehensive model training therefore requires large, diverse datasets covering relevant ranges of storage conditions, cultivars, and quality outcomes. Furthermore, the interpretability of ML models, particularly deep learning architectures, remains a challenge; developing explainable AI (XAI) techniques to elucidate which features most influence predictions can enhance trust and applicability in industrial settings. The accuracy of ML predictions is also contingent on cold‐chain data quality; sensor noise, missing values, and irregular sampling can degrade model performance. Systematic data preprocessing, feature selection, and robust cross‐validation strategies are therefore essential components of reliable ML modeling (Revelou et al. 2025; Tachie et al. 2023).

#### 5.4.4 Practical Applications and Future Directions

ANN and ML methodologies are already being deployed within intelligent cold‐chain systems to monitor storage conditions, forecast quality loss, and trigger alerts for potential temperature excursions. These systems support operational decision‐making by predicting how long a batch of frozen vegetables can maintain acceptable quality given its temperature history and current physicochemical state. Looking forward, integration of ML models with Internet of Things (IoT) sensor networks and predictive maintenance platforms promises more responsive cold‐chain management (Zhang et al. 2024). Hybrid models that combine mechanistic kinetic modeling with data‐driven ML predictions are also an active research direction, offering mechanistically informed yet flexible predictive tools capable of addressing the nonlinear, high‐dimensional nature of quality degradation in frozen tropical vegetables. ANN and machine learning approaches represent a powerful complement to classical kinetic and RSM models, enabling complex pattern recognition, real‐time monitoring, and adaptive shelf life prediction under realistic storage conditions. With continued advances in sensor technologies and data availability, ML is poised to play a central role in next‐generation quality management systems for frozen produce (Jiang, Zhang, et al. 2023; Fatorachian and Pawar 2025).

## 6 Strategies for Mitigating Color and Texture Degradation

**FIGURE 3:**
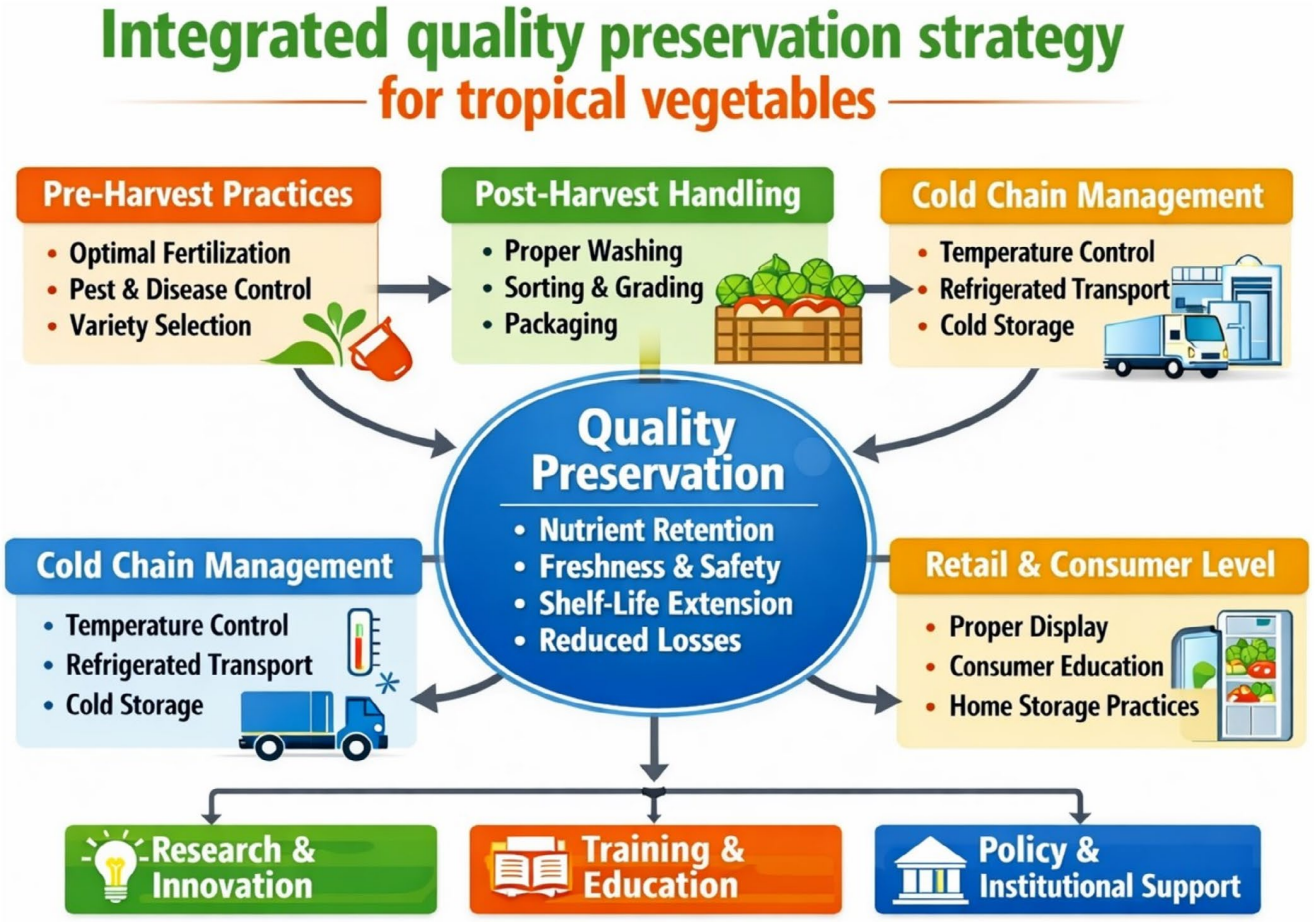
Integrated quality preservation strategy for frozen vegetables.

Effective preservation of color and texture in frozen tropical vegetables requires a multilayered mitigation framework that addresses the root causes of quality loss, namely residual enzymatic activity, oxidative reactions, moisture migration, and freeze‐induced structural damage. Rather than relying on a single control step, modern quality retention strategies combine optimized pretreatments, protective formulations, advanced freezing technologies, and barrier packaging systems to stabilize pigments and cellular architecture throughout storage (Figure 3). These interventions operate at different mechanistic levels: enzyme‐targeted treatments limit biochemical degradation, structural modifiers reinforce cell wall integrity, rapid and controlled freezing reduces mechanical injury, and atmosphere/packaging controls suppress oxidation and dehydration.

### 6.1 Optimized Blanching Treatments

Blanching prior to freezing primarily aims to inactivate endogenous enzymes responsible for pigment degradation (e.g., peroxidase, polyphenol oxidase) and cell wall breakdown, while minimizing detrimental effects such as nutrient leaching and textural weakening. The choice of blanching method profoundly influences heat transfer, tissue hydration, and enzyme inactivation kinetics, which in turn affect color and texture stability during freezing and storage (Ijod et al. 2025).

#### 6.1.1 Hot‐Water Blanching (HWB)

Hot‐water blanching (HWB) is the most traditional blanching technique, where vegetables are immersed in hot water at controlled temperatures (typically 80°C–95°C). It inactivates key enzymes and reduces microbial load, but heat and leaching of soluble compounds (e.g., chlorophylls, ascorbic acid) can compromise color and nutritional quality if parameters are not carefully optimized. Recent investigations demonstrate that short‐time HWB at optimized temperatures can balance enzyme inactivation with minimal pigment loss, but suboptimal conditions lead to excessive color leaching and textural degradation (Nambi et al. 2016). Recent investigations demonstrate that short‐time HWB at optimized temperatures can balance enzyme inactivation with minimal pigment loss, whereas suboptimal conditions lead to excessive color leaching and textural degradation. Hot‐water bath blanching at higher temperatures often inactivates chlorophyllase and oxidative enzymes while enhancing extractability of chlorophyll due to matrix changes, but longer exposures or higher temperatures also increase diffusion of pigments and vitamins out of the tissue (Managa et al. 2019). However, a limitation of HWB is its inefficiency in heat penetration, particularly for dense or large tissues, which may require increased time and thus lead to greater leaching and textural weakening. Recent research emphasizes dynamic monitoring of internal tissue temperature during HWB to ensure uniform blanching and avoid overprocessing (Xiao et al. 2017). Figure 3 illustrates the integrated mitigation strategies used to preserve color and texture in frozen tropical vegetables, including pretreatments, freezing technologies, and packaging interventions.

#### 6.1.2 Steam Blanching (SB)

Steam blanching (SB) employs saturated steam to deliver heat with minimal water contact, thereby reducing soluble nutrient loss. Mechanistically, SB induces rapid surface heating, denatures enzymes near the tissue interface, and preserves structural integrity better than prolonged HWB under comparable conditions. Several contemporary studies show that SB can achieve comparable enzyme inactivation to HWB while significantly reducing leaching of water‐soluble pigments and nutrients (Severini et al. 2016). In a systematic comparison of blanching methods applied to leafy and stem vegetables, studies reported that steam blanching at 95°C–98°C for short durations (1–2 min) effectively deactivated peroxidase and polyphenol oxidase with less reduction in chlorophyll content compared to HWB (Renate et al. 2025). Importantly, using steam minimized the loss of ascorbic acid and soluble sugars, leading to better color retention and firmness after freezing and storage. A key advantage of SB is its suitability for high‐throughput industrial lines, where heat transfer is rapid and consistent; however, steam intensity and exposure time must be carefully calibrated to avoid surface overheating and localized tissue collapse. Optimized SB can thus reduce ice‐nucleation heterogeneity and later ice crystal damage during freezing (Shi, Fu, et al. 2024).

#### 6.1.3 Microwave Blanching (MWB)

Microwave blanching (MWB) represents a nonconventional, volumetric heating approach where microwave energy interacts with polar molecules (mainly water) to generate heat rapidly throughout the tissue. This method promises reduced processing time, limited nutrient leaching, and better retention of thermolabile pigments compared with conventional heat conduction methods. Recent studies have found that microwave blanching (MW) markedly reduces blanching time for effective peroxidase inactivation in carrots compared with conventional hot‐water blanching, resulting in substantially higher retention of L‐ascorbic acid due to the shortened thermal exposure. However, MW treatment has also been reported to induce tissue damage and excessive softening as a consequence of rapid volumetric heating and internal pressure buildup, which can negatively affect texture despite improvements in nutrient retention (Orikasa et al. 2025). MWB also enhances cellular permeability in a controlled manner, which can facilitate more uniform freezing and smaller ice crystal formation due to improved moisture redistribution. However, MWB's primary challenge is avoiding localized overheating (“hot spots”), which can cause pitting and nonuniform softening. Strategies such as pulsed microwave application and optimization of microwave power–time combinations help mitigate these issues and enable broader industrial adoption (Saikumar et al. 2024).

### 6.2 Antioxidant and Anti‐Browning Treatments

To further mitigate color fading and texture deterioration in frozen tropical vegetables, a range of chemical and material‐based pretreatments can be applied in conjunction with blanching. These include the use of natural antioxidants and anti‐browning agents to delay oxidative pigment breakdown, calcium salts to reinforce cell wall structure and improve firmness, and emerging bio‐based coatings that serve as multifunctional barriers against oxygen, moisture, and enzymatic reactions. Collectively, these strategies address the underlying biochemical triggers of degradation, particularly pigment oxidation and enzymatic browning, and enhance structural stability, leading to improved quality retention during freezing and storage (Ungureanu et al. 2023).

#### 6.2.1 Natural Antioxidants

Pigment degradation and enzymatic browning are largely driven by oxidative processes and the action of enzymes such as polyphenol oxidase and peroxidase, which catalyze oxidation of phenolics into brown pigments (Ungureanu et al. 2023; Neri et al. 2020). A promising mitigation strategy involves the application of natural antioxidants, including plant extracts, phenolic compounds, and ascorbic acid derivatives, which can scavenge reactive oxygen species and suppress oxidative enzyme activity. These compounds not only delay pigment oxidation but can also act as reducing agents that convert intermediate quinones back to less reactive forms, thereby limiting browning reactions (Gupta et al. 2024). For example, research on fresh horticultural produce demonstrated that the incorporation of phenolic‐rich plant extracts or natural antioxidants into preservation systems significantly reduced browning indices and maintained color attributes during cold storage. Although these studies focus on fresh produce, the underlying mechanisms of antioxidant protection, ROS scavenging, chelation of prooxidant transition metals, and inhibition of oxidative enzymes are directly relevant to frozen systems where oxidative stress persists in unfrozen microenvironments within the tissue matrix (Pleșoianu and Nour 2022). This mechanistic linkage suggests that selecting natural antioxidants with strong radical scavenging capacity and enzyme inhibitory effects can be a valuable approach to protecting chlorophyll, carotenoids, and anthocyanins during extended frozen storage.

#### 6.2.2 Calcium Chloride Dips for Firmness Improvement

Calcium ions play a pivotal role in maintaining plant tissue integrity by interacting with pectic polysaccharides in the middle lamella to form “egg‐box” cross‐linked structures, which enhance cell–cell adhesion and reinforce cell walls. This ionic cross‐linking not only improves textural resilience but also influences biochemical responses that can indirectly affect pigment stability. For instance, exogenous CaCl_2_ treatments have been shown to upregulate phenylpropanoid metabolism and increase total phenolic content in fresh produce, thereby enhancing antioxidant capacity and reducing oxidative cleavage of pigments and membrane lipids (Saikaew et al. 2023). In frozen applications, calcium dips can strengthen tissues prior to freezing, reducing the extent of cell separation and structural collapse during ice crystal formation. A recent study on frozen mango pieces found that pretreatment with CaCl_2_ significantly retarded color changes during freezing and storage, maintained higher phenolic and flavonoid contents, and preserved overall antioxidant activity relative to untreated controls (Saikaew et al. 2023). This supports the dual role of calcium not only in textural enhancement but also in protecting against oxidative degradation pathways relevant to color stability.

#### 6.2.3 Emerging Bio‐Based Coatings

Bio‐based edible coatings and films represent a rapidly emerging strategy that integrates multiple protective functions forming semipermeable barriers, controlling gas and moisture exchange, and serving as carriers for antioxidants or antimicrobial agents. These coatings are typically derived from biopolymers such as polysaccharides (e.g., alginate, chitosan, and pectin), proteins (e.g., whey, soy), or composite materials, and can be formulated to improve barrier properties against oxygen and water vapor, reducing oxidative stress and dehydration during frozen storage (Popescu et al. 2022). Recent advances show that biopolymer‐based coatings significantly extend the postharvest quality of fruits and vegetables by decreasing respiration and transpiration rates, slowing color change, mitigating nutrient loss, and improving mechanical resilience. The addition of functional ingredients, such as natural antioxidants, organic acids, or essential oils, further enhances these effects by providing in situ scavenging of ROS and inhibition of discoloration pathways (Aljabary et al. 2025; Nabi et al. 2025).

In the context of frozen vegetables, coatings can act as a pre‐freezing barrier that reduces direct oxygen contact with sensitive pigments, limits moisture migration during freeze–thaw cycles, and generally moderates the tissue microenvironment to delay both enzymatic activity and physical degradation. Emerging research also highlights composite and nanostructured coatings with tailored barrier properties and controlled release profiles, which can sustain antioxidant delivery over prolonged storage, representing a promising area for future development in frozen produce quality management (Oliveira et al. 2025).

### 6.3 Advanced Freezing Technologies

Conventional air‐blast freezing at −18°C to −20°C remains the dominant industrial practice for frozen vegetables; however, its inherent limitations, particularly slow heat removal and extensive ice crystal growth, often lead to cellular disruption, pigment degradation, and irreversible texture softening. To overcome these constraints, advanced freezing technologies have been developed that aim to precisely control ice nucleation, crystal size, and phase transition dynamics. Among these, cryogenic freezing, pressure‐shift and isochoric freezing, and ultrasound‐assisted freezing have received increasing scientific attention due to their ability to significantly enhance microstructural preservation and quality retention in plant tissues (Grover and Negi 2023).

#### 6.3.1 Cryogenic Freezing

Cryogenic freezing employs extremely low temperatures using liquid nitrogen (−196°C) or liquid carbon dioxide (−78°C), enabling ultrarapid heat transfer rates and instantaneous surface freezing. This rapid thermal shock promotes the formation of a large number of small, uniformly distributed intracellular ice crystals, thereby minimizing mechanical rupture of cell walls and membranes. As a result, cryogenically frozen vegetables typically exhibit superior retention of cellular integrity, firmness, and natural color compared with conventionally frozen counterparts. Recent experimental work on vegetables and fruits has demonstrated that cryogenic freezing significantly reduces drip loss, preserves chlorophyll and carotenoid pigments, and maintains textural hardness after thawing, which is attributed to limited ice crystal growth and reduced solute concentration gradients within tissues (Jha et al. 2024). However, despite these quality advantages, the high operational costs, safety requirements, and nitrogen consumption limit large‐scale adoption, restricting cryogenic freezing primarily to high‐value or premium frozen products. Nonetheless, from a quality‐centric perspective, cryogenic freezing remains one of the most effective methods for preserving the structural and visual attributes of frozen vegetables (Vallespir et al. 2019).

#### 6.3.2 Pressure‐Shift Freezing and Isochoric Freezing

Pressure‐shift freezing (PSF) exploits the pressure dependence of the water–ice phase transition, where water remains liquid at subzero temperatures under high pressure and freezes rapidly upon pressure release. This mechanism allows for simultaneous and uniform ice nucleation throughout the product, resulting in fine ice crystals and reduced cellular damage (Li et al. 2022). Studies on plant‐based systems have shown that PSF effectively preserves cell wall structure, firmness, and pigment stability, outperforming traditional freezing in terms of microstructural integrity (Bilbao‐Sainz et al. 2020). Isochoric freezing, a more recent and conceptually distinct approach, involves freezing food in a constant‐volume (rigid) system, where pressure builds naturally during ice formation. Unlike conventional freezing, isochoric systems suppress ice recrystallization and reduce freeze‐concentration effects without requiring mechanical pressure application. Emerging evidence suggests that isochoric freezing can maintain textural firmness and color stability in fruits and vegetables over extended storage periods, even at moderately higher subzero temperatures. While still largely confined to laboratory‐scale research, both PSF and isochoric freezing represent paradigm‐shifting technologies with strong potential for reducing freeze‐induced damage in sensitive tropical vegetables (Dhanya et al. 2023).

#### 6.3.3 Ultrasound‐Assisted Freezing

Ultrasound‐assisted freezing (UAF) introduces high‐intensity ultrasonic waves into the freezing medium or product, inducing acoustic cavitation and micro‐streaming effects. These phenomena enhance heat transfer and promote controlled ice nucleation, leading to smaller ice crystals and faster freezing rates. Unlike cryogenic or pressure‐based systems, UAF can be integrated into existing freezing infrastructures, making it an attractive hybrid technology. Recent studies on vegetables have demonstrated that ultrasound application during freezing improves texture retention, color stability, and water‐holding capacity, while reducing thawing loss and cellular disruption (Wu et al. 2022). Importantly, UAF also limits enzymatic activity by accelerating phase transition and reducing the time tissues spend in the critical temperature zone (−1°C to −5°C), where enzymatic and oxidative reactions are most active. Despite its promise, optimization of ultrasound frequency, power density, and treatment duration remains essential, as excessive acoustic energy may induce localized structural damage. Overall, ultrasound‐assisted freezing represents a technically versatile and scalable strategy for enhancing frozen vegetable quality when carefully controlled (Aghajani et al. 2023).

#### 6.3.4 Critical Perspective

Advanced freezing technologies fundamentally alter ice formation dynamics, offering targeted solutions to the microstructural and biochemical challenges associated with frozen vegetable storage. Cryogenic freezing provides unmatched quality retention but faces economic barriers, while pressure‐based systems and isochoric freezing offer novel thermodynamic control with strong future potential. Ultrasound‐assisted freezing bridges the gap between innovation and practicality by enhancing freezing efficiency within conventional systems. Strategic integration of these technologies with optimized pretreatments may define the next generation of high‐quality frozen tropical vegetables.

### 6.4 Advanced Packaging and Atmosphere Control

Maintaining quality during frozen storage is not only a function of how vegetables are processed and frozen, but also of the packaging system used to isolate them from the external environment. Packaging for frozen tropical vegetables must address challenges including moisture migration, oxygen ingress, freezer burn, enzymatic oxidation, and sublimation, all of which contribute to color and texture loss. Advanced packaging strategies such as customized atmosphere packaging (CAP), vacuum packaging, and multilayer high‐barrier films modify the microenvironment around the product, thereby slowing quality deterioration and extending shelf life (Dwibedi et al. 2024; Mengozzi et al. 2024).

#### 6.4.1 Customized Atmosphere Packaging

Customized Atmosphere Packaging (CAP), including Modified Atmosphere Packaging (MAP), intentionally alters the gaseous composition within a sealed package to create conditions that slow deteriorative reactions. In frozen produce, reducing oxygen concentration and increasing inert gases such as nitrogen or elevated carbon dioxide can inhibit oxidative pigment degradation, reduce enzymatic browning, and suppress moisture loss. Although most MAP research has focused on fresh and chilled vegetables, its principles are directly relevant to frozen systems where residual oxygen drives slow oxidation even at subzero temperatures (Saikaew et al. 2025).

In MAP, high‐barrier films help stabilize the modified gas mix, maintaining low O_2_ levels that slow oxidation and enzymatic pathways that degrade chlorophyll and other pigments, while elevated CO_2_ can inhibit aerobic degradation reactions. Recent critical reviews emphasize that MAP can extend overall shelf life and preserve sensory quality by mitigating oxidation and microbial growth, suggesting that tailored gas compositions may also support superior color and texture retention in frozen tropical vegetables when integrated with freeze‐stable packaging structures (Czerwiński et al. 2021). Implementation challenges include selecting appropriate gas mixtures for specific crops (respiring vs. non‐respiring tissue) and ensuring that packaging materials maintain gas barriers over extended storage. Nevertheless, CAP represents a powerful atmosphere control tool that complements blanching and advanced freezing technologies to maintain export‐grade quality.

#### 6.4.2 Vacuum Packaging

Vacuum packaging removes air and therefore oxygen from the headspace around the food prior to sealing, creating an anaerobic environment that directly addresses two major quality‐degrading pathways: oxidation of sensitive pigments and moisture migration leading to freezer burn. By eliminating virtually all atmospheric oxygen, vacuum packaging slows oxidative deterioration of chlorophylls and carotenoids, which are prone to breakdown even at low temperatures when oxygen is present. Although most vacuum packaging research has focused on meat and high‐fat foods, the fundamental mechanism inhibition of oxidation by oxygen exclusion directly applies to frozen vegetables where oxidative browning and membrane lipid peroxidation contribute to quality loss (Wagoner et al. 2022). A study on vacuum‐packed fresh corn demonstrated significantly improved sensory and physicochemical stability under prolonged storage compared with non‐vacuum controls, highlighting the protective benefits of oxygen elimination for texture and color preservation. For frozen tropical vegetables, combining vacuum sealing with robust heat‐seal multilayer packaging enhances barrier performance and minimizes sublimation and freezer burn. However, vacuum packaging alone does not control residual moisture or internal humidity, so it is often used in conjunction with high‐barrier films and desiccants or humidity absorbers to further mitigate moisture‐related degradation (Li et al. 2023).

#### 6.4.3 Multilayer Films With High Barrier Packaging

High‐barrier packaging films leverage multilayer structures composed of different polymers and coatings to achieve exceptional barriers against oxygen, moisture, and volatile migration. Typical high‐barrier films combine layers such as PET, EVOH, polyamide (PA), and sealant layers (e.g., PE/LLDPE) to create a composite structure that dramatically reduces oxygen transmission rate (OTR) and water vapor transmission rate (WVTR) compared with single‐layer films. These film architectures maintain controlled atmospheres within packages and slow oxidative and moisture‐induced quality losses throughout frozen storage (Xie 2023).

Scientific evaluations of barrier film performance demonstrate that multilayer composites significantly reduce gas ingress and moisture exchange, directly contributing to slowed oxidation, retention of color indices, and improved texture relative to less protective packaging (Mengozzi et al. 2024). For example, composite films featuring EVOH layers combined with nylon and PE have been shown to outperform monolayer films by extending freshness and sensory quality by over 30%–40% in comparative barrier studies (Yang et al. 2025). In practice, multilayer high‐barrier packaging is often combined with MAP or vacuum techniques to create synergistic effects, where low permeability films stabilize modified atmospheres or near‐vacuum conditions, thereby maximizing protection against oxidation and freezer burn while also preserving texture and nutritional quality. Emerging innovations include biopolymer‐based multilayer composites and active barrier layers integrated with oxygen scavengers or humidity absorbers, which further enhance protective performance while aligning with sustainability goals (Bauer et al. 2022).

### 6.5 Novel Additives and Natural Preservatives

Growing consumer demand for clean‐label and minimally processed foods has intensified research into novel additives and natural preservatives capable of mitigating color and texture degradation in frozen vegetables. Unlike conventional synthetic preservatives, these agents function through antioxidant activity, enzyme inhibition, membrane stabilization, and water‐binding mechanisms, while aligning with sustainability and regulatory acceptance. Recent studies increasingly emphasize their compatibility with freezing processes and long‐term frozen storage (Lemoni et al. 2025).

#### 6.5.1 Plant‐Derived Antioxidants and Polyphenolic Extracts

Natural antioxidants derived from plant sources including phenolic acids, flavonoids, and tannins are widely recognized for their strong antioxidant activity, which helps mitigate oxidative degradation of pigments during storage. Plant polyphenols such as catechins in green tea have documented free‐radical scavenging abilities and can interact with oxidative enzymes like polyphenol oxidase (PPO) and peroxidase (POD), reducing oxidative pathways that lead to color loss in plant tissues (Yan et al. 2020; Amarowicz and Pegg 2019).

For instance, treatments with tea polyphenols have been shown to slow chlorophyll degradation and delay yellowing in leafy vegetables by enhancing overall antioxidant capacity and bolstering both enzymatic and nonenzymatic antioxidant defenses during storage. While specific studies on frozen conditions at −18°C are limited in the open literature, the demonstrated ability of tea polyphenols to retard pigment loss under controlled storage suggests similar protective mechanisms would operate in frozen matrices due to reduced oxidative activity at low temperatures (Pang et al. 2025).

Similarly, phenolic extracts rich in rosmarinic acid (RA) a major antioxidant compound in herbs like rosemary and lemon balm are recognized for their high radical‐scavenging capacity and potential to stabilize pigments prone to oxidation. Rosmarinic acid and related phenolic compounds have been identified as effective natural antioxidants in food systems, capable of limiting oxidative deterioration of color‐sensitive compounds such as carotenoids and anthocyanins by interacting with reactive species and reducing oxidative chain reactions (Parveen et al. 2025; Khojasteh et al. 2020). Together, these plant‐derived antioxidants align with clean‐label demands and can confer synergistic effects with mild pretreatments (e.g., blanching) by reducing the severity of thermal processing needed to inactivate degradative enzymes while helping maintain color and quality during extended frozen storage.

#### 6.5.2 Organic Acids and Enzyme‐Inhibiting Natural Compounds

Organic acids such as ascorbic acid, citric acid, and malic acid are widely recognized for their dual role as antioxidants and enzyme inhibitors. Recent work has demonstrated that pre‐freezing dipping treatments with ascorbic–citric acid blends effectively reduced enzymatic browning and chlorophyll degradation in frozen green vegetables by lowering tissue pH and chelating metal ions required for PPO activity. Importantly, these acids exhibited minimal negative impact on texture when optimized at low concentrations (Yang et al. 2024). Beyond classical organic acids, emerging studies have highlighted the effectiveness of natural enzyme inhibitors such as phytic acid and plant‐derived sulfhydryl compounds, which selectively suppress PPO and POD activity without causing excessive pigment leaching. Their application in frozen vegetables has been associated with improved visual quality and delayed onset of discoloration during extended storage (Fang et al. 2022).

#### 6.5.3 Polysaccharides and Hydrocolloids

Natural polysaccharides, including alginate, pectin, chitosan, and cellulose derivatives, are increasingly investigated as multifunctional additives that enhance both color and texture stability. These biopolymers act as cryoprotectants, reducing ice recrystallization, limiting moisture migration, and reinforcing cell wall integrity. Recent findings indicate that low‐molecular‐weight chitosan coatings applied prior to freezing significantly reduced firmness loss and drip loss in frozen vegetables by stabilizing membrane structures and reducing enzymatic softening (Zhang et al. 2025). Pectin‐based formulations have also gained attention for their ability to interact with calcium ions in the middle lamella, thereby strengthening cell‐to‐cell adhesion and reducing texture collapse during frozen storage. When combined with calcium salts, these systems form semirigid networks that resist mechanical damage caused by ice crystal growth (Saikaew et al. 2023).

#### 6.5.4 Future Potential

While novel additives and natural preservatives show strong promise for improving frozen vegetable quality, their effectiveness is highly matrix‐dependent and influenced by factors such as vegetable type, pretreatment conditions, freezing rate, and storage temperature. A critical challenge remains the optimization of dosage and application method to balance quality preservation with sensory neutrality and cost‐effectiveness. Future research should focus on synergistic combinations of natural preservatives with advanced freezing and packaging technologies, as well as mechanistic studies linking molecular interactions to macroscopic quality outcomes. Integrating these additives into predictive quality models may further enhance their industrial applicability in frozen tropical vegetable systems.

## 7 Industrial Implications and Export Relevance in Storage

Frozen tropical vegetables occupy a strategically important position in global agri‐food trade, particularly for exporting regions in South and Southeast Asia, Africa, and Latin America. However, export success is highly contingent on the ability to maintain visual quality, texture integrity, and functional stability throughout extended frozen storage and long‐distance cold‐chain logistics. Color and texture degradation directly influence market acceptance, regulatory compliance, and economic competitiveness, making quality preservation a decisive industrial concern rather than a purely technological one (Wang et al. 2025; Abdel et al. 2025).

### 7.1 Consumer Perception and Market Demand

Consumer perception of frozen vegetables is predominantly driven by visual appearance and textural fidelity after thawing, which act as immediate proxies for freshness and nutritional value. In export markets, especially in Europe, North America, and East Asia, consumers increasingly expect frozen vegetables to closely resemble fresh produce in terms of greenness, firmness, and structural integrity. Even minor discoloration (e.g., chlorophyll loss or browning) or excessive softening after cooking can lead to product rejection, negative brand perception, and reduced repeat purchases (Huang et al. 2022; Wang et al. 2025). Importantly, consumer expectations are evolving alongside demand for clean‐label, minimally processed, and sustainably preserved products. This trend places additional pressure on exporters to limit severe thermal treatments or synthetic additives while still achieving enzyme inactivation and structural stability. Consequently, technologies that mitigate color and texture degradation without compromising perceived naturalness are gaining strong commercial relevance.

### 7.2 Quality Standards and Trade Barriers

International trade in frozen vegetables is governed by stringent quality, safety, and labeling standards, which function as both safeguards and nontariff trade barriers. Regulatory frameworks such as Codex Alimentarius standards, European Union regulations, and FDA guidelines emphasize consistency in color, absence of enzymatic browning, acceptable texture after thawing, and stable sensory attributes over declared shelf life. Failure to meet these standards, particularly regarding visual uniformity and textural performance, can result in shipment rejection, downgrading of product category, or mandatory relabeling. For tropical vegetables, which are often less familiar to importing markets, deviations from expected quality norms are scrutinized more strictly. This places exporters at a disadvantage if color fading, freezer burn, or excessive softening occurs during prolonged storage or temperature abuse in transit (Ferro et al. 2015).

### 7.3 Challenges for Tropical Vegetable Exporters

Exporters of tropical vegetables face a unique set of structural and technological challenges that exacerbate quality degradation risks. These include high intrinsic moisture content, fragile cell wall architecture, and elevated enzymatic activity, all of which increase susceptibility to freezing‐induced damage. In addition, inconsistent access to rapid freezing technologies, temperature‐stable cold chains, and advanced packaging materials further compounds degradation during export logistics. Another major challenge lies in cold‐chain discontinuity, particularly during transshipment, port handling, and last‐mile distribution. Even short‐term temperature fluctuations can accelerate ice recrystallization, enzymatic reactivation, and pigment oxidation, leading to irreversible quality loss. For small and medium exporters, limited capital investment capacity restricts adoption of advanced freezing or predictive quality monitoring systems, creating a technological gap between developing and developed exporting nations.

### 7.4 Economic Viability and Competitiveness

From an economic perspective, maintaining color and texture stability directly influences product yield, market price, and profit margins. Quality degradation results in increased trimming losses, downgraded product classification, shorter commercial shelf life, and higher rates of customer rejection. These factors collectively erode competitiveness in price‐sensitive global markets. Conversely, investment in optimized blanching, advanced freezing technologies, high‐barrier packaging, and predictive quality modeling can significantly enhance export viability by extending shelf life and reducing quality variability (Ran et al. 2025; Iyer and Robb 2025). Although such investments increase initial processing costs, they often yield favorable cost–benefit outcomes through reduced waste, improved brand reputation, and access to premium markets. For tropical vegetable exporters, long‐term competitiveness increasingly depends on transitioning from cost‐driven production models to quality‐driven, technology‐enabled export strategies (Ugrinov et al. 2024).

## 8 Future Research Directions

Despite significant progress in understanding color and texture degradation in frozen tropical vegetables, several knowledge gaps and technological limitations remain. Addressing these gaps is essential for improving product stability, export competitiveness, and sustainability in global frozen food systems. Future research should move beyond empirical observations toward mechanistically grounded, system‐level, and data‐driven approaches.

### 8.1 Molecular‐Level Understanding & Freeze–Thaw Dynamics

Current studies largely describe macroscopic quality changes (color loss, softening) without fully elucidating the molecular and subcellular mechanisms underlying pigment transformation, membrane destabilization, and cell wall disassembly during frozen storage. Advanced analytical tools such as omics‐based approaches (proteomics, metabolomics) and in situ spectroscopic techniques should be employed to track enzyme–substrate interactions, pigment derivatives, and structural polymers at low temperatures (Vyse et al. 2020). This is particularly important for tropical vegetables, whose biochemical composition and enzyme profiles differ substantially from temperate crops. Most laboratory studies rely on isothermal storage conditions, which do not adequately represent real‐world cold‐chain environments characterized by temperature fluctuations and intermittent thawing events. Future research should focus on dynamic freeze–thaw modeling, incorporating realistic logistics scenarios, to quantify cumulative damage from ice recrystallization, enzymatic reactivation, and moisture migration. Coupling experimental data with time–temperature integrators and digital cold‐chain monitoring tools will enhance the predictive accuracy of quality degradation models.

### 8.2 Data‐Driven and Crop‐Specific Research

The application of machine learning, deep learning, and hybrid kinetic–AI models in frozen vegetable quality prediction is still at an early stage. Future research should focus on developing interpretable AI models that link input variables (temperature history, enzyme activity, moisture state, and packaging properties) with mechanistic quality outcomes. Integration of large‐scale industrial datasets, including sensor‐based cold‐chain data, will be crucial for transitioning these models from academic tools to decision‐support systems for processors and exporters (Zhang et al. 2024). A major limitation in the literature is the extrapolation of findings from temperate vegetables to tropical species. Given the structural fragility, higher enzymatic activity, and distinct pigment composition of tropical vegetables such as taro stolon, lablab bean, okra, and yardlong bean, future research must adopt crop‐ and cultivar‐specific frameworks. Comparative studies across varieties will help identify intrinsic traits linked to freeze tolerance and guide breeding or selection programs aimed at improved frozen stability.

### 8.3 Integration of Advanced Strategies

Although individual mitigation strategies (e.g., blanching optimization, cryogenic freezing, antioxidant treatments, high‐barrier packaging) have been studied extensively, their synergistic interactions remain poorly understood. Future work should prioritize multifactorial experimental designs that integrate processing, formulation, freezing, and packaging variables. Such holistic approaches are necessary to identify optimal combinations that maximize color and texture retention while minimizing energy input, processing severity, and additive load. Future research should also address the environmental and economic sustainability of frozen vegetable preservation. This includes optimizing freezing energy consumption, developing biodegradable or recyclable high‐barrier packaging materials, and valorizing processing by‐products rich in natural antioxidants or cryoprotective compounds. Life cycle assessment (LCA) approaches should be integrated with quality studies to balance shelf life extension against environmental impact.

Finally, there is a critical need for translational research that bridges laboratory findings with industrial implementation. Pilot‐scale trials, techno‐economic analyses, and validation under commercial storage and export conditions are essential to ensure that proposed innovations are scalable, cost‐effective, and regulatory‐compliant. Strengthening collaboration between researchers, processors, exporters, and policymakers will be key to accelerating adoption and maximizing the global impact of advances in frozen tropical vegetable preservation.

## 9 Conclusions

Although freezing remains the most effective preservation method for tropical vegetables, significant color and texture degradation continues during frozen storage due to ice crystal‐induced cellular damage, enzymatic activity, and oxidative reactions. This review highlights that these mechanisms are strongly influenced by processing history, storage temperature stability, and intrinsic tissue structure. Classical kinetic and Arrhenius‐based models provide valuable insight into degradation behavior and shelf life estimation, while advanced modeling approaches, including response surface methodology and machine learning, offer improved predictive capability under complex and dynamic storage conditions. Recent technological advances, including optimized blanching, natural antioxidant systems, calcium‐based firmness enhancers, advanced freezing technologies, and high‐barrier packaging, demonstrate strong potential to mitigate quality losses when strategically integrated. For tropical vegetable exporters, particularly those operating in long and variable cold chains, adopting predictive quality tools alongside targeted technological interventions is critical for maintaining market competitiveness. Future research should focus on validating integrated modeling–technology frameworks under real‐world conditions to ensure consistent quality retention and sustainable expansion of frozen tropical vegetable markets.

## Author Contributions

**Muhammad Muntasir Mahmud:** methodology, data curation, investigation, writing – original draft, formal analysis, software. **Md. Hassan Bin Nabi:** software, data curation, formal analysis, writing – review and editing. **Abid Hassan Arnab:** software, formal analysis, visualization, data curation. **Wahidu Zzaman:** conceptualization, project administration, supervision, writing – review and editing, resources, data curation. **Md. Suhel Mia:** software, validation, formal analysis, writing – review and editing.

## Ethics Statement

The authors have nothing to report.

## Conflicts of Interest

The authors declare no conflicts of interest.

## Data Availability

The data that support the findings of this study are available from the corresponding author upon reasonable request.
